# Novel ecto-tagged integrins reveal their trafficking in live cells

**DOI:** 10.1038/s41467-017-00646-w

**Published:** 2017-09-18

**Authors:** Clotilde Huet-Calderwood, Felix Rivera-Molina, Daniel V. Iwamoto, Emil B. Kromann, Derek Toomre, David A. Calderwood

**Affiliations:** 1 Department of Pharmacology, Yale University School of Medicine, 333 Cedar Street, New Haven, Connecticut 06520 USA; 2 Department of Cell Biology, Yale University School of Medicine, 333 Cedar Street, New Haven, Connecticut 06520 USA; 3 Department of Biomedical Engineering, Yale University, 333 Cedar Street, New Haven, Connecticut 06520 USA

## Abstract

Integrins are abundant heterodimeric cell-surface adhesion receptors essential in multicellular organisms. Integrin function is dynamically modulated by endo-exocytic trafficking, however, major mysteries remain about where, when, and how this occurs in living cells. To address this, here we report the generation of functional recombinant β1 integrins with traceable tags inserted in an extracellular loop. We demonstrate that these ‘ecto-tagged’ integrins are cell-surface expressed, localize to adhesions, exhibit normal integrin activation, and restore adhesion in β1 integrin knockout fibroblasts. Importantly, β1 integrins containing an extracellular pH-sensitive pHluorin tag allow direct visualization of integrin exocytosis in live cells and revealed targeted delivery of integrin vesicles to focal adhesions. Further, using β1 integrins containing a HaloTag in combination with membrane-permeant and -impermeant Halo dyes allows imaging of integrin endocytosis and recycling. Thus, ecto-tagged integrins provide novel powerful tools to characterize integrin function and trafficking.

## Abstract (web summary)

Integrins are cell-surface adhesion receptors that are modulated by endo-exocytic trafficking, but existing tools to study this process can interfere with function. Here the authors develop β1 integrins carrying traceable tags in the extracellular domain; a pH-sensitive pHlourin tag or a HaloTag to facilitate dye attachment.

## Introduction

The ability of cells to sense and adhere to the surrounding extracellular matrix (ECM) is essential for multicellular life. Integrins, a family of heterodimeric αβ adhesion receptors, enable this by binding specific ECM ligands with their ectodomains and associating with a wide range of cytoskeletal and signaling proteins through their cytoplasmic tails, permitting bidirectional transmembrane communication that is essential for cell adhesion, migration, differentiation, and survival^1–3^. Integrin-mediated adhesion and signaling is regulated by diverse factors including conformational rearrangements that alter the affinities of integrins for their extracellular ligands, clustering of integrins and their intracellular binding partners into cytoskeletal-associated adhesions, as well as the dynamic endocytosis, sorting, and exocytosis of integrins themselves^1, 2, 4, 5^.

Although much is known about integrins at the atomic level (i.e., the molecular basis for ligand binding and the conformational domain rearrangements involved in integrin activation^6–9^), fundamental insight into the spatial and temporal control of integrin functions at the cellular level is critically lacking. Specifically, where and when integrins become engaged/disengaged to enable physiological responses such as adhesion, migration, differentiation, and survival, and how spatial and temporal dysregulation of these processes contributes to disease, remain to be fully elucidated. Integrin trafficking, as a way to control integrin surface levels via exocytosis, endocytosis, and recycling, has received considerable interest^4, 5, 10^, especially as alterations in integrin trafficking have been shown to promote invasion and cancer metastasis^4, 11–13^. Many molecular adapters involved in membrane trafficking have been found to regulate integrin surface levels and to affect integrin-mediated activities, with some adapters shown to directly bind integrin subunits^4, 5, 10, 14, 15^. Although biochemical assays such as cell-surface biotinylation or integrin labeling with ligand or antibodies have allowed measurement of integrin internalization and recycling rates, fully understanding how integrin trafficking is orchestrated and its role in cell physiology and pathology requires sophisticated microscopy tools designed to follow specific pools of integrins in live cells. To date, direct visualization of integrin exocytosis has not been possible but integrin endocytosis has been imaged using either integrin subunits fused to a cytoplasmic fluorescent protein (cyto-tagged), or indirect integrin labeling with specific ligands or antibodies^15–17^. Together with FRAP and photoconversion techniques, cyto-tagged integrins have been powerful tools to visualize integrin internalization and turnover^18, 19^. Photoactivation in Total Internal Reflection Fluorescence Microscopy (TIRFM) has been used to localize the sites of integrin internalization^18^ and to determine to which portion of the cell α5β1 is preferentially delivered^11^. However, cyto-tagged integrins have a number of shortcomings. First, the inaccessibility of a cytoplasmic tag to the extracellular compartment precludes the use of affinity or enzymatic tags for selective and covalent surface labeling. Second, the insensitivity of a cytoplasmic tag moiety to the extracellular environment prevents the use of pH-sensitive fluorophores to discriminate whether the integrins are at the cell surface or in endomembranes. Third, there are valid concerns about the impact of the cytoplasmic tag on the binding of the numerous cytoplasmic partners^19–22^ to the relatively short (20–70 amino acids) cytoplasmic tail of integrin subunits.

As a consequence, we set out to design functional recombinant β1 integrins containing an accessible and traceable extracellular tag (ecto-tag). The main challenge was to identify, within the multi-domain structure of the β1 integrin ectodomain, a suitable tag insertion site that would affect neither the overall folding of each subdomain, nor heterodimerization with the α integrin subunit, nor the ligand-binding activity and specificity. Moreover, because it is generally thought that integrin activation and ligand binding trigger substantial structural rearrangements, including extension of the integrin from a bent to extended conformation and reorientation of specific domains within the complex^3, 6, 23^, the ecto-tag should not impact the equilibrium between active and inactive conformations so as to preserve integrin function.

Here we report the successful generation of functional ecto-tagged β1 integrins with various genetic and chemical-genetic fluorescent tags (GFP, pHluorin, or Halo tags) inserted into an exposed loop in the hybrid domain. Using pHluorin as a pH-sensitive ecto-tag provided the first live images of fusion of β1 integrin-rich vesicles with the plasma membrane, and suggests preferential vesicular fusion in the vicinity of focal adhesions (FAs). Using HaloTag as an enzymatic ecto-tag that covalently binds synthetic chemical ligands^24^, we selectively labeled cell-surface and intracellular β1 integrins with different fluorophores and dynamically followed them simultaneously to illustrate β1 integrin internalization and trafficking in cells. We propose that the ecto-tagged integrins described herein will provide new and powerful tools for live imaging of integrins in cells, and will allow for detailed spatiotemporal dissection of integrin trafficking.

## Results

### Insertion of a tag in the β1 integrin ectodomain

**Fig. 1:**
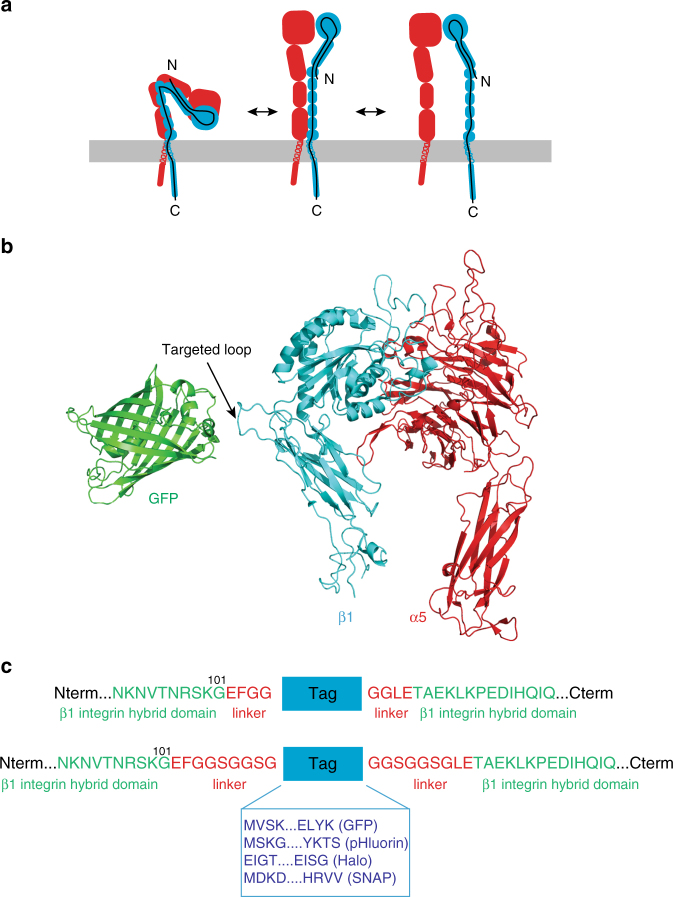
Design of an ecto-tagged β1 integrin. **a** Cartoon of the conformational changes in the integrin heterodimer during integrin activation; α subunit is depicted in *red*, β subunit in *blue*, and the *black line* represents the β subunit polypeptide chain. **b** Ribbon diagram of the crystal structures of the α5β1 integrin head piece (PDB: 3VI4) and GFP (PDB: 1GFL). The hybrid domain loop into which ecto-tags were inserted is indicated. **c** Zoom-in on the amino acid sequence of human ecto-tagged β1 integrins at the tag insertion site. Each ecto-tag (GFP, pHluorin, Halo, and SNAP, in *blue*, N- and C-terminal sequences specified) was inserted into the hybrid domain of human β1 integrin between residues Gly101 and Tyr102 (in *green*). Linkers of 4 or 9 amino acids (in *red*) were added on each side of the tag to facilitate cloning and provide flexibility

To generate new tools that facilitate imaging of integrins and their trafficking, we set out to introduce tags into the extracellular domains of the widely expressed β1 integrins. Integrin β subunits contain 8 extracellular domains and notably the three N-terminal domains are nested within one another (Fig. 1a). Several factors restrict the number of suitable sites for placement of a large tag such as GFP, Halo or SNAP: (i) the large aforementioned nested N-terminal domains, (ii) the presence of an N-terminal signal peptide that gets cleaved off during polypeptide maturation, (iii) the need to retain the ability to pair with integrin α subunits to bind ligand, and (iv) the need to accommodate the large-scale conformational rearrangements thought to occur during integrin activation. Thus, finding a suitable non-perturbing site is far from trivial.

To identify suitable tagging sites we examined published crystal structures of headpiece fragments of α5β1^25^, and both headpiece and full-length ectodomains of αvβ3^26–29^ and αIIbβ3^6, 30–34^ alone or in complex with inhibitors or ligands. We sought a surface loop that was consistently exposed in full-length bent structures as well as active headpiece structures, and which was far enough away from the ligand-binding site that it would be unlikely to clash with bound ligand. We selected a long exposed loop (β1 residues 92–114) between β strands X and A (following the nomenclature of Xiong et al.^26^) in the hybrid domain (Fig. 1b). This loop was exposed in the crystal structures of αIIbβ3 and αvβ3 integrins. Furthermore, although this loop is shorter in β2 integrins it is located in a similar exposed position in structures of the full-length bent inactive αxβ2^35^. Notably, the loop lies within the hybrid domain, which undergoes substantial motion during activation and ligand binding, increasing the risk that tags may alter the activation/inactivation equilibrium. Indeed, the allosteric inhibitory β1 antibody SD/19 binds to this loop and a SD/19 Fab was co-crystalized with the α5β1 headpiece^25^. However, SD/19 also makes contacts with the β subunit A domain and probably inhibits ligand binding by wedging between the β A-domain and the hybrid domain, preventing the swing-out of the hybrid domain required for ligand binding^25^. We therefore hypothesized that inserting a tag with a flexible linker into this exposed loop (residues 92–114) would be accommodated.

To generate ‘ecto-tagged’ β1 integrins, we introduced unique restriction sites into a human β1 cDNA between the codons for Gly101 and Tyr102 and inserted PCR-amplified GFP, pHluorin, SNAP, or Halo sequences at these sites, along with 4- or 9-amino acid flexible linkers on both sides (Fig. 1c). As controls we used untagged human β1 integrin and a previously described β1 integrin containing a GFP fused to the C-terminus of the cytoplasmic tail^21, 36^. All expression constructs were introduced into pLENTI lentiviral expression vectors for transduction of target cells.

### Ecto-tag β1 integrins are expressed at the cell surface

**Fig. 2:**
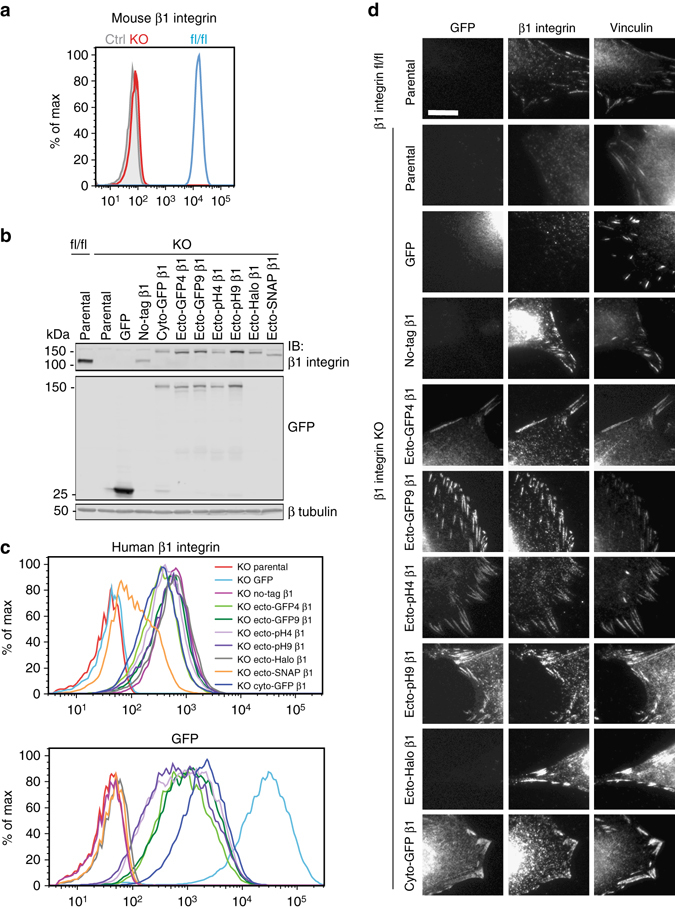
Ecto-tagged β1 integrins translocate to the cell surface and target to FAs. **a** Flow cytometric analysis of cell surface levels of mouse β1 integrin in β1 integrin fl/fl (*blue peak*) and KO fibroblasts (*red peak*), and control unstained KO fibroblasts (*gray peak*). **b** Immunoblot detection of endogenous and ectopic β1 integrins in lysates of 100,000 fl/fl and KO fibroblasts using a pan-β1 integrin antibody (*top panel*) and an anti-GFP antibody (*middle panel*). Tubulin was used as a loading control (*bottom panel*). Uncropped blots are available in Supplementary Fig. 10. **c** Flow cytometric analysis of cell surface levels of human β1 integrins (*top panel*) and GFP fluorescence (*bottom panel*) in parental and reconstituted β1 integrin KO fibroblasts. **d** Microscopy images of parental and reconstituted fl/fl and KO fibroblasts showing GFP epifluorescence (*left column*), β1 integrin immunofluorescence with 9EG7 antibody (*center column*), and vinculin immunofluorescence (*right column*). *Scale bar*, 10 μm

To test if ecto-tagged β1 integrins are functional, we reconstituted β1 integrin knockout (KO) mouse fibroblasts with ectopically expressed ecto-tagged human β1 integrins, or for comparison, untagged (no-tag) or cytoplasmically-tagged (cyto-GFP) β1 integrins. Flow cytometry confirmed that the clonal line of CRE-infected β1 integrin KO cells lacks surface mouse β1 integrin, when compared with parental β1 integrin floxed fibroblasts (fl/fl) and control unstained KO fibroblasts (Ctrl; Fig. 2a). Following stable lentiviral-mediated transduction of the β1 integrin KO fibroblasts with our human β1 integrin expression constructs, or with GFP alone, we assessed β1 expression by immunoblotting whole-cell lysates using an antibody that detects both mouse and human β1 integrin (Fig. 2b). As expected, β1 integrin was detected in parental β1 integrin fl/fl fibroblasts but not in β1 integrin KO cells. Using the same anti-β1 integrin antibody we detected all ectopically expressed human β1 integrins at their expected molecular weight. Furthermore, immunoblotting with an anti-GFP antibody detected GFP- and pHluorin-tagged integrins. Importantly, in most cases the ectopically expressed integrins appear to be intact with little or no degradation fragments evident. Although the anti-β1 integrin antibody bound both mouse and human β1 integrins, it may not do so equally, making it impossible to directly compare expression levels of the ectopically expressed human integrins and endogenous mouse integrins.

To test whether tagged integrins were expressed at the cell surface, we assessed human β1 integrin levels in reconstituted fibroblasts by flow cytometry with the anti-human β1 integrin antibody P5D2. As expected, parental fl/fl and KO cells as well as KO cells expressing GFP alone lacked human β1 (Fig. 2c, *upper panel*). Cell-surface β1 integrins were detected in all reconstituted lines. Notably, with the exception of cells expressing ecto-SNAP β1 integrin where surface β1 integrin expression was low and heterogeneous, all other β1-expressing lines appeared as single populations with relatively homogeneous β1 integrin surface levels. Moreover, cells reconstituted with GFP or with β1 integrin carrying GFP or pHluorin tags exhibited GFP fluorescence (Fig. 2c, *lower panel*), indicating that the fluorescent protein was properly folded when inserted into β1 integrin ectodomain. We thus conclude that β1 integrins with a GFP, pHluorin, or Halo tag inserted into the βX-βA loop of the hybrid domain are expressed and are effectively targeted to the plasma membrane. Notably, the length of the spacer (4 or 9 amino acids) had little impact on expression or targeting. While SNAP-tagged β1 integrin is expressed, little reaches the plasma membrane and we suspect that defective folding or aggregation leads to its accumulation in the endoplasmic reticulum (ER). For this reason the ecto-SNAP β1 integrin was not characterized further.

### Ecto-tag β1 integrins target to focal adhesions

In adherent fibroblasts, integrins cluster at cell-matrix adhesion sites—most notably FAs—where they co-localize with a range of cytoplasmic and cytoskeletal proteins. We therefore investigated whether ecto-tagged β1 integrins localized to FAs in reconstituted β1 integrin KO fibroblasts plated on fibronectin (FN)-coated coverslips. Immunofluorescence using the conformation-specific antibody 9EG7, which recognizes active β1 integrins^37^, showed that all ectopically expressed human β1 integrins (no-tag, ecto-tag, and cyto-GFP) co-localized with the FA marker vinculin, just like endogenous mouse β1 integrin in parental fl/fl cells (Fig. 2d). We did not observe any obvious effect of the tags on FA size or number, which were highly variable in our fibroblast population. As expected, no β1 integrin signal was detected in parental KO cells or KO cells expressing GFP alone, although these cells still formed vinculin-rich FAs, presumably via endogenous αvβ3 or αvβ5 integrins^38^. GFP fluorescence could also be detected in FAs in cells expressing human β1 integrins carrying a GFP or pHluorin tag, indicating that their fluorescent tag was functional.

The preceding data show that ecto-tagged integrins localize normally to FAs. However, to assess whether they exhibit defective maturation or accumulate in the ER we performed immunofluorescence with the activation-insensitive anti-β1 antibody AIIB2^39^. AIIB2 staining confirmed that ectopically expressed human β1 integrins (no-tag, ecto-tag, and cyto-GFP) co-localize with FA markers but also revealed more intracellular staining than seen with the activation-specific anti-β1 antibody 9EG7, consistent with some ER accumulation (Supplementary Fig. 1a). This intracellular staining was evident with all exogenously expressed integrins, regardless of whether they contained an ecto-tag, cyto-tag, or no-tag, indicating that the ecto-tag does not cause a maturation defect. To test whether the intracellular staining is related to integrin expression levels we varied the quantity of virus used to drive β1 expression. We observed that lowering expression levels reduced the intracellular signal while retaining adhesion targeting for all β1 integrins tested, tagged or not (Supplementary Fig. 1b). Consistent with this, flow cytometry showed that diluting the virus reduced cell-surface expression of both no-tag and ecto-GFP4 β1 integrins proportionately, and also reduced total GFP signal for ecto-GFP4 β1 (Supplementary Fig. 1c). However, the ratio of cell surface ecto-GFP4 β1 to total GFP signal increased at lower viral titers indicating that, at lower integrin expression levels, a greater percentage is present on the cell surface (Supplementary Fig. 1d). Together these data show that ecto-tagged integrins traffic efficiently to the cell surface where they form adhesions, and that when expressed at appropriate levels they do not aberrantly accumulate inside the cell.

To examine a larger spectrum of adhesions, we imaged β1 KO fibroblasts stably co-expressing the adhesion marker paxillin-mCherry and various ecto-tagged or no-tag β1 integrins. Cells were plated on FN-coated glass-bottomed plates and adhesions examined 1 h (Supplementary Fig. 2a) or 24 h (Supplementary Fig. 2b) after plating. In all cases, co-localization of ecto-tagged and untagged integrins with paxillin-mCherry was evident in nascent adhesions (Supplementary Fig. 2a), mature FAs, and fibrillar adhesions (Supplementary Fig. 2b). Ecto-tagged integrins also co-localized with paxillin-mCherry in FAs in cells plated on type I collagen (Supplementary Fig. 2c) and ecto-Halo β1 integrins localized in FA in HeLa cells also expressing endogenous β1 integrins (Supplementary Fig. 2d). Thus, inserting an ecto-tag into human β1 integrin hybrid domain did not impair targeting to a spectrum of adhesions on a range of integrin substrates.

### Ecto-tag β1 integrins rescue adhesion in β1-null fibroblasts

**Fig. 3:**
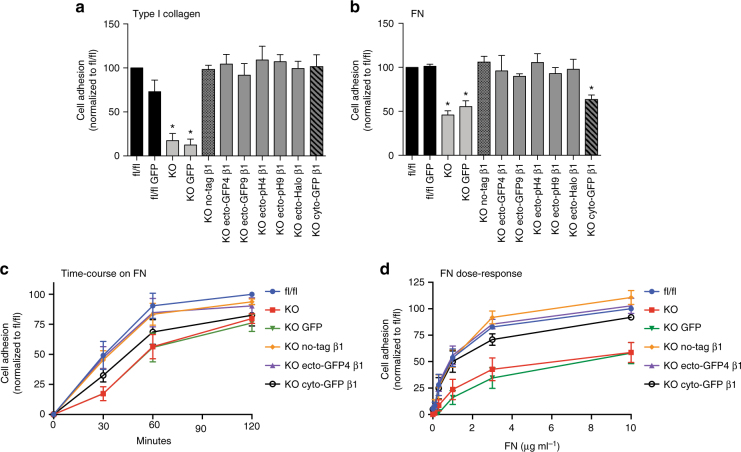
Ecto-tagged β1 integrins rescue the adhesion defect of β1 integrin KO fibroblasts. **a** Adhesion of parental and reconstituted β1 integrin fl/fl and KO fibroblasts 2 h after plating on wells coated with 3 μg ml^−1^ type I collagen. **b** Adhesion of parental and reconstituted β1 integrin fl/fl and KO fibroblasts 1 h after plating on wells coated with 3 μg ml^−1^ FN. **c** Time course of adhesion of parental and reconstituted β1 integrin fl/fl and KO fibroblasts on wells coated with 3 μg ml^−1^ FN. **d** Dose-response adhesion assay of parental and reconstituted β1 integrin fl/fl and KO fibroblasts for 1 h to wells coated with increasing concentrations of FN. In all experiments, cell adhesion was measured by the absorbance at 570 nm after Crystal Violet staining. Data is shown as mean ± SEM from three independent experiments. In **a**, **b**, statistical analysis was performed using one-way repeated measures ANOVA with Dunnett post hoc test. Each column was compared to fl/fl and *p < 0.05

Our results indicate that ecto-tagged integrins are expressed, traffic to the plasma membrane, and localize to FAs, suggesting that these integrins are functional. To test this further we assessed whether they could restore normal adhesion in β1 integrin KO cells. β1-containing integrins are major collagen receptors in fibroblasts, and consistent with this, compared to parental β1 integrin fl/fl fibroblasts, β1 integrin null fibroblasts exhibited severely impaired adhesion to plates coated with 3 μg ml^−1^ type I collagen (Fig. 3a). When β1 integrin KO fibroblasts were reconstituted with untagged, ecto- or cyto-tagged human β1, adhesion to collagen was fully restored to levels seen with β1 integrin fl/fl fibroblasts, while GFP alone had no effect (Fig. 3a). Similar results were observed with FN-coated plates; however, in this case KO cells exhibited only ~50% inhibition in adhesion, and the β1 cyto-GFP construct was unable to fully rescue adhesion (Fig. 3b). It is likely that, in the absence of α5β1, KO cells adhere via αvβ3 or αvβ5 integrins^38^, explaining the partial adhesion defect. To examine this further, we performed time-course (Fig. 3c) and dose-response (Fig. 3d) adhesion assays on FN comparing the ability of no-tag, ecto-GFP4, and cyto-GFP β1 integrins to rescue cell adhesion. Results showed that ecto-GFP4 β1 and no-tag β1 were practically undistinguishable at all time points and all FN doses tested, while cyto-GFP β1 integrin exhibited only a very small temporary lag in adhesion. Thus, insertion of an ecto-tag in β1 integrin hybrid domain preserves function.

### Ecto-tag β1 integrins are active

**Fig. 4:**
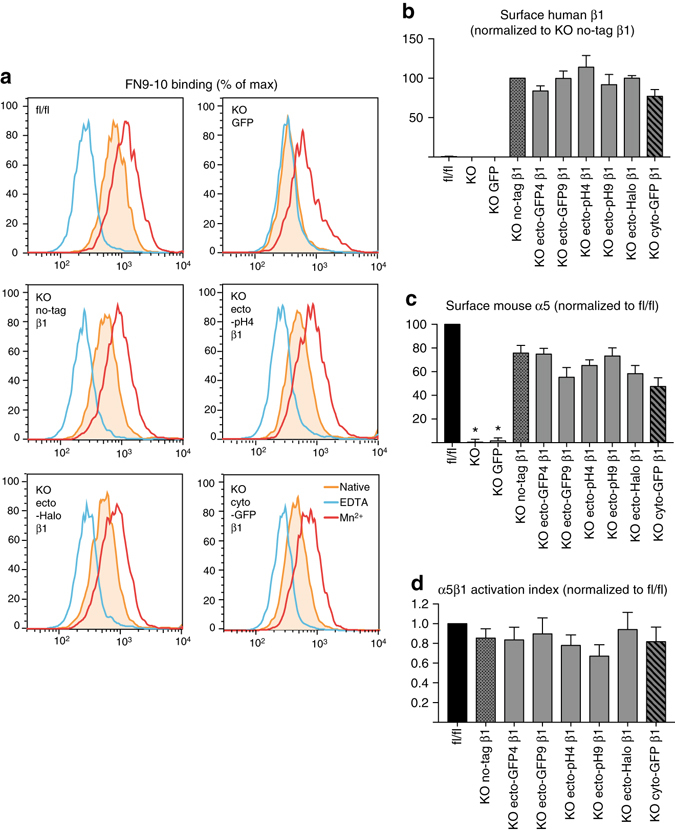
Ecto-tagged β1 integrins bind soluble ligand, restore surface levels of endogenous α5 integrins, and display normal activation indices. **a** Flow cytometry histograms showing binding of soluble FN9-10 to parental and a subset of reconstituted KO fibroblasts, in native conditions (filled *orange peak*), EDTA-inhibited conditions (*blue peak*), or Mn^2 +^ -treated conditions (*red peak*). Full data set in Supplementary Fig. 3a. **b**, **c** Quantification of surface levels of human β1 (**b**) and mouse α5 integrins (**c**) measured by flow cytometry on parental and reconstituted β1 integrin fl/fl and KO fibroblasts. **d** Activation index of surface α5β1 integrins on parental and reconstituted β1 integrin fl/fl and KO fibroblasts, calculated as (FN9-10 binding in native conditions—FN9-10 binding in EDTA-inhibited conditions)/surface levels of α5 integrins. All data **b**–**d** is shown as mean ± SEM from four independent experiments. Statistical analysis was performed using one-way repeated measures ANOVA with Dunnett post hoc test. Each column was compared to KO no-tag β1 and *p < 0.05

One concern with inserting tags in the hybrid domain is that it may alter integrin activation, perhaps by stabilizing extended active structures leading to hyper-activation of integrins. The similar adhesion of cells expressing untagged and ecto-tagged integrins (Fig. 3) suggests that this is not the case, but to directly test this question we assessed binding of a soluble FN fragment containing FN type III repeats 9 and 10 (FN9-10) in a well-established flow cytometric assay^40^. Fl/fl fibroblasts displayed clear binding of FN9-10 (Fig. 4a, *orange shaded peak*) that was both inhibited by ethylenediaminetetraacetic acid (EDTA; blue peak) and stimulated by Manganese (Mn^2+^, red peak). Specific binding of FN9-10 (native binding—binding in EDTA) was greatly reduced in GFP-expressing β1 KO cells, as binding in native condition was very similar to binding in EDTA-treated condition (Fig. 4a). Notably, while expression of GFP alone had no effect, specific binding of soluble FN9-10 was restored when β1 integrin KO cells were reconstituted with any of the human β1 integrins (no-tag, cyto-GFP, and ecto-tags) as evidenced by the higher FN9-10 binding in native vs. EDTA-treated conditions (Fig. 4a and Supplementary Fig. 3a). Moreover, FN9-10 binding in reconstituted cells was enhanced by manganese.

Hence our data indicate that introducing a GFP, pHluorin, or Halo tag in β1 integrin hybrid domain is compatible with ligand binding and does not interfere with its regulation by EDTA or manganese. To quantify integrin activation we measured specific binding and normalized it to integrin expression levels. Although all the reconstituted cells exhibited comparable levels of human β1 integrin (Fig. 4b), due to antibody specificity, we were unable to directly compare levels of human β1 integrins in reconstituted cells with levels of endogenous mouse β1 integrin in fl/fl cells. To circumvent this problem, we measured surface levels of endogenous mouse α5 integrin as a means to assess surface α5β1 levels. Mouse α5 integrin, an obligate β1 integrin partner, was present at the surface of β1 integrin fl/fl cells but not at the surface of β1 integrin KO cells (Fig. 4c), in agreement with previous data showing that α and β integrin subunits have to heterodimerize in order to be transported to the cell surface. Importantly, in β1 integrin KO cells, α5 surface levels were rescued by ectopic expression of any of the human β1 integrins (Fig. 4c). Notably, α5 surface levels did not quite reach the levels observed in β1 integrin fl/fl cells, and we speculate that this could be either due to a suboptimal pairing of mouse α5 with human β1 integrin subunits or due to differences in α5 levels between the clonal β1 integrin KO line and the parental polyclonal β1 integrin fl/fl cell population. Nonetheless, when surface levels of α5 were used to calculate the activation index of α5β1 (specific binding normalized by integrin expression), we found that cells reconstituted with no-tag β1, cyto-GFP β1, or ecto-tag β1 all had similar activation indices, only slightly below that of parental fl/fl cells (Fig. 4d). In addition, the activation-specific hybrid domain-binding antibody HUTS-4^41^ bound equivalently to both untagged and ecto-tagged integrins (Supplementary Fig. 3b). Therefore we conclude that α5β1 integrin dimers containing ecto-tag β1 integrins are not hyperactivated when compared with either α5β1 integrins containing untagged human β1 or with endogenous mouse α5β1 integrins, suggesting that the insertion of the tag into the hybrid domain does not substantially interfere with the processes of activation and inactivation.

### Live imaging of pHluorin-β1 integrin exocytosis

Having established the functionality of ecto-tagged β1 integrins, we sought to take advantage of the pH sensitivity of pHluorin fluorescence to visualize integrin exocytosis in live cells. Based on our experience with previously generated ecto-pHluorin-tagged membrane proteins^42–44^, we predicted that (1) ecto-pHluorin β1 integrins would emit little fluorescence in secretory vesicles due to the quenching of ecliptic pHluorin at acidic pH, and that (2) during exocytosis, ecto-pHluorin β1 integrin-loaded secretory vesicles fusing with the plasma membrane would expose their cargo to the neutral extracellular environment (pH~7.4) resulting in a burst of green fluorescence. This burst of fluorescence is expected to be transient due to the rapid diffusion of the pHluorin cargo in the plasma membrane.

**Fig. 5:**
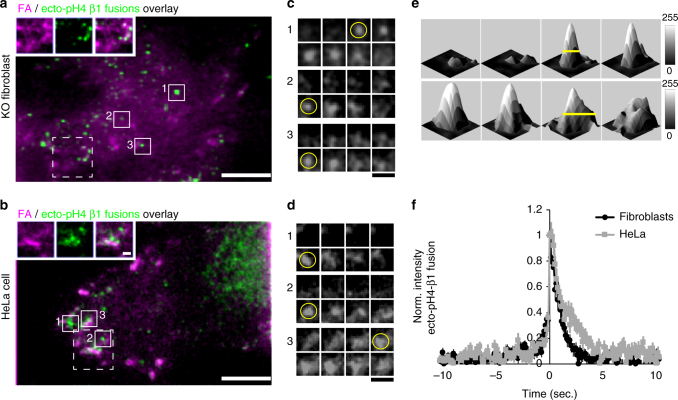
Visualization of ecto-pHluorin-β1 integrin exocytosis in live cells by TIRFM. **a**, **b** Overlay images of ecto-pH4 β1 integrins prior to photobleaching to mark the footprint of FA (*magenta*) in KO fibroblasts reconstituted with ecto-pH4 β1 integrin (**a**) or HeLa cells overexpressing ecto-pH4 β1 integrin (**b**), and, the maximum projection of ecto-pH4 β1 integrin fusion events after photobleaching (fusions, *green label*; Supplementary Movies 1 and 2). *Scale bar*, 10 µm. *Top left corner* insets show fusion events (*green*) in close proximity to FA (*magenta*) (**a**, **b**; *dashed line square*). *Scale bar*, 2 µm. **c**, **d**, Galleries of single ecto-pH4 β1 integrin fusion events over time (*solid squares* on **a**, **b**; 0.25 s between images). *Yellow circle* denotes event in which the vesicle signal intensity of the ecto-pH4 β1 integrin fluorescence rapidly intensifies, likely due to de-acidification upon opening of the fusion pore. *Scale bar*, 2.5 µm. **e** Surface plot analysis of a fusion event showing the increase on the full-width half maximum (FWHM) of the signal peak over time, consistent with bona fide full vesicle fusion (*yellow lines*; 0.25 s between plots). **f** Temporal alignment of fusion events detected on reconstituted KO fibroblasts (*black line*) or HeLa cells (*gray line*) cells showed the expected changes in ecto-pH4 β1 integrin during fusion. Data are shown as mean ± SEM for 67 and 44 events, respectively (2 cells for each)

Live β1 integrin KO fibroblasts re-expressing ecto-pHluorin β1 integrin were imaged by TIRFM to visualize pHluorin-β1 integrin exocytosis at the plasma membrane-matrix interface (Fig. 5a)^43, 44^. Live-cell TIRFM imaging confirmed our observation in fixed cells that pHluorin-β1 integrin localized to FAs (Fig. 5a, *magenta label*). Similar results were obtained in HeLa cells stably expressing pHluorin-β1 integrin on a wild-type β1 integrin background (Fig. 5b), supporting that our constructs are effective in different cell types and can be applied even in the presence of endogenous β1 integrin. Photobleaching of the background surface signal (Supplementary Fig. 4) allowed us to visualize for the first time pHluorin-β1 integrin exocytosis events (Fig. 5a, b, *green label*; Supplementary Movies 1–2). Critically, pHluorin-β1 integrin cargo incorporated and dispersed into the plasma membrane in a manner that shows a clear signature of exocytic fusion^43^: (1) a rapid increase in signal when a pHluorin cargo-loaded vesicle de-acidified (Fig. 5c, d; *yellow circles*), (2) the full-width half maximum (FWHM) of the signal peak increased over time (Fig. 5e, *yellow lines*), and (3) the average vesicular profile of multiple fusion events (n = 44–67), which were time-aligned to fusion, showed the expected intensity spread during exocytosis^43^ (Fig. 5f). Notably, this approach allowed us to visualize pHluorin-β1 integrin exocytosis events, which appeared to occur preferentially near FAs (Fig. 5a, b, *dashed line square*; inset).

**Fig. 6:**
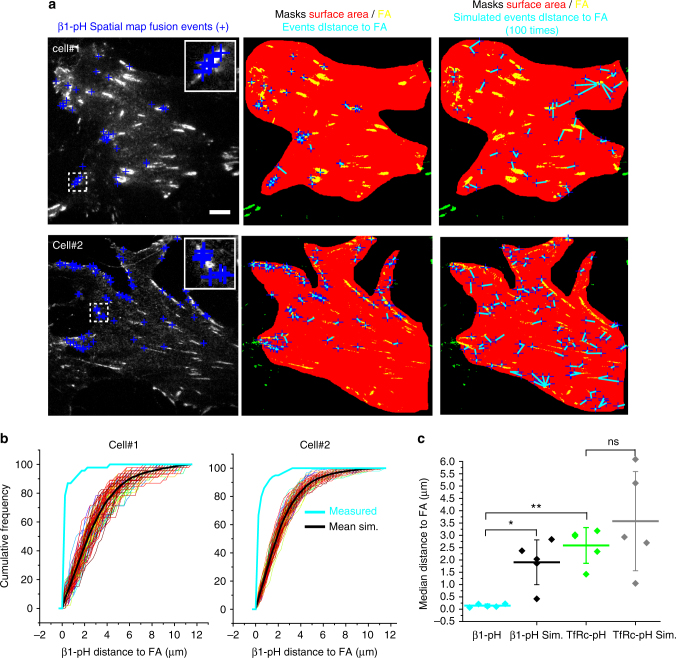
Spatial distribution analysis of ecto-pH4 β1 integrin fusion events supports direct integrin delivery to FAs. **a** *Left panel*, Distribution of fusion events (*blue crosses*) and FAs (*white*) detected by TIRFM in two KO fibroblasts reconstituted with ecto-pH4 β1 integrins (β1-pH). *Center panel*, the distance of fusion events to the nearest FA (*yellow mask*) was measured (*cyan lines*). *Right panel*, the distance of randomly simulated events around the cell surface (*red mask*) to the nearest FA were measured (*cyan lines*). *Scale bar*, 10 µm. **b** Cumulative frequency charts for the two cells demonstrating the difference in distance to FA between the measured data (*cyan line*) and 100 simulations (individual simulation, *color lines*; mean, *black line*). **c** Quantification of the median distance to FAs for β1-pH or TfRc-pH fusion events, and the simulations (β1-pH Sim and TfRc-pH Sim) performed in the same cells. The scatter-box graph shows the mean ± SD for 5 cells and the respective simulation data. Statistical analysis was performed using a two samples Student’s *t*-test. *p < 0.05, **p < 0.01

Integrin association with specific vesicular adapters of the trafficking machinery suggests that their delivery to particular areas on the cell is spatially regulated, and we therefore sought to rigorously test the hypothesis that integrin exocytosis might preferentially occur in close proximity to existing FAs. To test this hypothesis, we generated spatial maps of integrin fusion events from 5 cells (Supplementary Fig. 5a) and overlaid them relative to a FA map (Fig. 6a, *yellow mask*). Here too, fusion events appeared to cluster in close proximity to FAs (Fig. 6a, *inset blue crosses*). To statistically test this concept, Monte Carlo simulations were used to randomly assign the same number of events throughout the cell surface area imaged by TIRFM (Fig. 6a, *red mask*)^45, 46^. Using a custom algorithm we computed the distance of each event to the closest FA in both the real data and after 100 Monte Carlo simulations (Fig. 6b), which showed that pHluorin-β1 integrin (β1-pH) fusion events were highly clustered near FAs (Fig. 6b, *cyan line*) while simulated events were more randomly scattered on the cell surface (Fig. 6b, *black line*). Interestingly, only a subset of the FAs appeared to be targeted by fusion events, even in these non-polarized cells. When combined from 5 different cells, measurements of the median distance to FAs was strikingly different between β1-pH fusion events and simulated events (Fig. 6c), with β1-pH showing an average distance of <0.25 μm compared to ~2 μm in the simulations—strongly supporting a model of preferential delivery of integrins to FAs.

For comparison and to test the possibility that the clustering of integrin fusion events at FAs could be due to a distance artifact (the cell membrane is closer to glass at FAs than in the rest of the cell^47, 48^), we analyzed the spatial distribution of transferrin receptor-pHluorin (TfRc-pH) fusion events from 5 cells expressing the FA maker Paxillin-mCherry. Fusion events exhibited the expected signature of pHluorin-tagged cargo delivered to the cell surface and were distributed along the ventral cell surface (Supplementary Fig. 5b). Mapping analysis of the distance of TfRc-pH fusion events to FAs showed that these events occur over 10-fold further away from FAs than the β1-pH events (Fig. 6c; Supplementary Fig. 6; *cyan* vs. *green line*). In fact, the distance to FAs measured for TfRc-pH fusion events was not significantly different from that of randomly occurring simulated events in the same cells (Fig. 6c; Supplementary Fig. 6; *green* vs. *gray line*). Together, these findings support that exocytosis of β1-integrin, but not a general recycling cargo, shows a high spatial selectivity for fusion at FAs.

Our analysis of pHluorin-tagged β1 integrin exocytosis provides the first direct, quantitative evidence for targeted delivery of integrin-rich vesicles to FAs. The mechanism by which this spatial targeting is achieved is unknown, but prior reports have shown accumulation of integrins in FAs at the leading edge of migrating cells in a phosphoinositide-regulated exocyst-complex-dependent manner^49^, and our ecto-tagged integrins provide a powerful tool for future investigation of this process.

### Dynamic live imaging of Halo-β1 integrin endocytosis

**Fig. 7:**
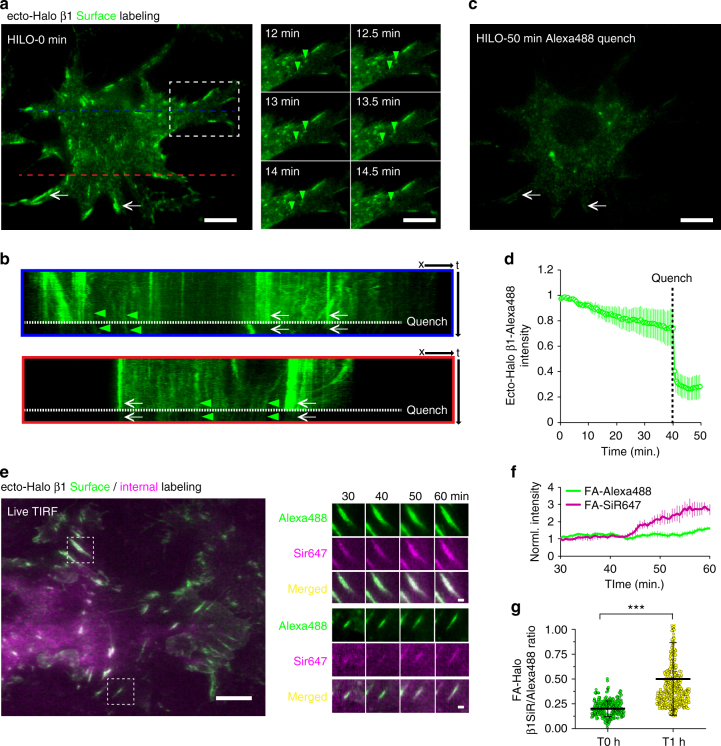
Selective surface and internal labeling of ecto-Halo β1 integrins demonstrates integrin endocytosis in live cells. **a** Labeling of surface ecto-Halo β1 integrins in reconstituted KO fibroblasts with Alexa488 Halo ligand and imaging by HILO TIRFM immediately after labeling (0 min). *Scale bar*, 15 µm. *Right panel*, movement of Alexa488-positive vesicle-like structures (*dashed line rectangle*, *green arrowheads*). *Scale bar*, 10 µm. **b** Kymograph of two regions of the cell (*blue* and *red lines*) illustrating the dynamics of Alexa488-positive structures before (*green arrowheads*) and after the addition of Alexa488 quencher antibody (*white arrows*). **c** Residual Alexa488 signal after quenching, imaged by HILO TIRFM 50 min post labeling (*white arrows*). *Scale bar*, 15 µm. **d** quantification of normalized ecto-Halo β1 Alexa488 intensity before and after quenching. The line graph shows the mean ± SD of the normalized intensity for 5 cells. **e** Overlay image of KO fibroblasts reconstituted with ecto-Halo β1 integrins, after sequential labeling with Alexa488 and SiR647 Halo ligands and imaged by TIRFM. *Scale bar*, 15 µm. Images on the right show the time series of two FAs (*dashed line square*). *Scale bar*, 2.5 µm. **f** Line graph showing the temporal changes in SiR647 and Alexa488 Halo signals in FAs measured by TIRFM during a 30 min period (mean ± SD for 4 FAs). **g** Scatter-box graph showing the mean ± SD for the ratio of SiR647/Alexa488 Halo signals of 388 and 523 FAs imaged by TIRFM 0 h or 1 h after incubation at 37 °C on fixed samples (6 cells per conditions). Statistical analysis was performed using a two samples Student’s *t*-test and ***p < 0.001

Integrin endocytosis and recycling is another important aspect of integrin traffic. When assessed biochemically using reversible cell-surface biotinylation, Ecto-GFP4 β1 (Supplementary Fig. 7a, b) and ecto-Halo β1 (Supplementary Fig. 7c, d) internalized at rates comparable to untagged β1 integrins. To assess ecto-Halo β1 endocytosis by live cell imaging we took advantage of the interchangeable chemical-genetic labeling technology of HaloTag, a modified haloalkane dehalogenase that covalently binds synthetic small-molecule ligands which can be coupled to fluorophores or other affinity tags^24, 50, 51^. Importantly, both membrane-permeant and -impermeant fluorescent Halo substrates are commercially available (e.g., Promega). First, we specifically and selectively labeled surface ecto-Halo β1 integrins in live β1 integrin KO fibroblasts with the cell-impermeant HaloTag Alexa Fluor 488 Ligand (Fig. 7a; Supplementary Fig. 8a). Determining the levels of labeled ecto-Halo β1 integrins endocytosis in live cells by TIRFM imaging can be a challenge since: (1) TIRFM illumination only excites the labeled integrins close to the glass surface, and (2) the high levels of labeled integrins at the surface can mask the detection of dimmer internal structures. To overcome these challenges (1) we used highly inclined and laminated optical sheet (HILO) illumination^52^ to obtain deeper images of labeled ecto-Halo β1 integrins in cells and with a better signal/noise ratio than spinning disk confocal and (2) we quenched surface fluorescence by treating cells with an antibody against Alexa488 to unmask internalized labeled integrins^53^ (Fig. 7a, c; Supplementary Movie 3). The HILO illumination approach allowed us to visualize the appearance of Alexa488-positive vesicle-like structures inside the cells (Fig. 7a; *right panel*, *green arrow heads*), which displayed curvilinear tracks and ‘squiggles’ as seen by kymographs (Fig. 7b, *green arrowheads*; focal adhesions (white arrows) hardly moved). After imaging for 40 min, the surface fluorescence was quenched in living cells with the anti-Alexa488 antibody, as evidenced by the strong decrease in signal at the cell surface and at FAs (Fig. 7b, c; *white arrows*). Residual Alexa488 signal remaining after quenching was in small vesicle-like structures inside the cell, which continued to move (Fig. 7b, c). Quantification of the normalized integrin labeling intensity of 5 cells before vs. after quenching (Fig. 7d) supports that 20–25% of the surface ecto-Halo β1 integrin signal had endocytosed in 40 min, a value that agrees with the internalization rate of Halo-tagged β1 integrins measured biochemically (Supplementary Fig. 7c, d). Further supporting that a portion of ecto-Halo β1 was endocytosed, we observed partial co-localization with pulse-labeled (30 min) transferrin-Alexa568 (Tf568; Supplementary Fig. 9a, b).

We then explored the potential of using HaloTag ligands with distinct properties to label various pools of β1 integrins in cells. We used a sequential labeling strategy to separately label surface and internal pools of ecto-Halo β1 integrins in live cells. We first incubated ecto-Halo β1 integrin-reconstituted KO fibroblasts with the cell-impermeant Alexa488 Halo Ligand and then with the cell-permeant SiR647 Halo ligand, with the expectation that prior incubation with Alexa488 Halo ligand would saturate surface Halo sites, resulting in intracellular-only labeling with SiR647 Halo ligand. Indeed, the Alexa488 Halo ligand labeled surface ecto-Halo β1 integrins present in FAs (Supplementary Fig. 9c, *green label*, *green arrowheads*) and stained the ventral and dorsal surface of the cell (Supplementary Fig. 9e). In contrast, the SiR647 Halo ligand did not label FAs (Supplementary Fig. 9c T0h, *magenta label*), rather it stained an internal pool of β1 integrins (Supplementary Fig. 9e), mainly in the ER, the Golgi, and endosomes (Supplementary Fig. 8b). We then examined the long-term changes in subcellular localization of these two pools of β1 integrins. 16 h after sequential Halo labeling, Alexa488-positive intracellular vesicular structures were visible by confocal microscopy (Supplementary Fig. 9d, *green label, right bottom panel*), indicating that some surface β1 integrins had been internalized. After 16 h, SiR647-labeled integrins co-localized with Alexa488-labeled integrins in FAs as well as in intracellular structures (Supplementary Fig. 9d, *yellow arrowheads*), indicating that some β1 integrins from the intracellular pool at T_0_ trafficked to the cell surface and incorporated into FAs. Line scans and Pearson’s correlation analysis confirmed an increase in surface SiR647 staining and co-localization of Alexa488 and SiR647 after 16 h (Supplementary Fig. 9e, f). Interestingly, FAs were still labeled with Alexa488 16 h after labeling (Supplementary Fig. 9d), indicating either a very low internalization rate for surface β1 integrins or alternatively a high recycling rate and recruitment into de novo assembled FAs.

To test whether rapid β1 integrin recycling takes place, we undertook dual-color labeling, with a sequential 15 min pre-labeling and live-cell imaging by TIRFM (Fig. 7e–g; Supplementary Movie 4). As expected, at early time points, FAs were mainly labeled by ecto-Halo β1 Alexa488, but over a time period of ~ 30 min FAs were additionally labeled with an intracellular pool of ecto-Halo β1 SiR647 (Fig. 7e, f). Quantification of several hundred FAs showed that the labeling with SiR647 increased ~2.5-fold over 1 h, supporting rapid delivery of an internal integrin pool (Fig. 7g). Together, these results demonstrate the feasibility of using ecto-Halo β1 integrins in combination with various Halo ligands to selectively label surface and internal pools of integrins, and image and quantify their endocytosis and trafficking in live cells.

## Discussion

Endocytosis and exocytosis regulate integrin-mediated adhesion and signaling^4, 10^. Consequently there is considerable interest in understanding integrin trafficking, with development of innovative tools to follow integrin endocytosis^11, 15–18^. However, current tools have not allowed direct visualization of exocytosis. Here we describe the design, validation, and application of novel recombinant ecto-tagged β1 integrins with tags in an exposed extracellular loop of the β1 integrin hybrid domain. We establish that the insertion of GFP, the pH-sensitive GFP variant pHluorin, or the enzymatic tag Halo at this site does not disrupt β1 integrin function. Using ecto-pHluorin β1 integrin to visualize integrin exocytosis in live cells we reveal preferential delivery of β1 integrins to FAs. Furthermore, we show that ecto-Halo β1 integrins permit visualization of integrin endocytosis in live cells. We anticipate that future development of cleavable membrane-impermeant Halo ligands should allow discrimination between biosynthetic and recycling pools of integrins and detailed analysis of internalization or recycling kinetics. We initially focused on the β1 subunit because it is widely expressed and can pair with a large number of different α subunits, potentially allowing us to investigate a range of integrin heterodimers. However, we note that, as different heterodimers use distinct trafficking pathways^15^, the promiscuity of the β1 subunit makes discrimination of the routes and kinetics of specific heterodimers difficult to isolate, and in the future specific ecto-tagged α subunits may complement the use of ecto-tagged β subunits. Nonetheless, we anticipate that ecto-tagged β1 integrins will provide an important toolkit for integrin research and will greatly facilitate detailed investigation of the integrin trafficking in live cells and potentially in vivo.

## Methods

### Constructs

Plasmid DNA encoding human β1 integrin C-terminally fused to GFP (pcDNA3-β1 cyto-GFP)^21^ was provided by Dr. Anthony Koleske (Yale University). An untagged human β1 expression construct (pcDNA3 no-tag-β1) was generated by subcloning from pcDNA3-β1 cyto-GFP. To generate ecto-tagged β1 integrin pcDNA3 expression constructs, we used QuikChange Mutagenesis to insert a GAATTCCTCGAG sequence introducing unique EcoRI and XhoI sites into the ecto-domain coding region of pcDNA3 no-tag-β1 integrin between codons encoding Gly101 and Tyr102 (Supplementary Table 1). We PCR-amplified eGFP from the pEGFP-C2 vector (Clontech), ecliptic pHluorin from the sspH-mSmo vector^54^, HaloTag from C-Halo vector (Promega) and SNAP from pSNAP β Adrenergic Rc vector (New England BioLabs), using primers that added a 5′ linker containing an EcoRI site and a 3′ linker containing a XhoI site along with a spacer sequence (Supplementary Table 1). Each ecto-tag was then inserted into the β1 integrin ectodomain using the ectopic XhoI-EcoRI sites. We generated ecto-GFP and ecto-pHluorin β1 integrin constructs containing either 4 amino acid flanking linkers (ecto-GFP4 and ecto-pH4) or 9 amino acid flanking linkers (ecto-GFP9, ecto-pH9), while ecto-Halo and ecto-SNAP constructs were generated only with 9 amino acid flanking linkers (see Fig. 1 for exact sequence information). A paxillin-mCherry expression vector was built by PCR and cloning using a *Gallus Gallus* Paxillin-DsRed vector (a gift from Christopher Turner, Upstate Medical University) and pmCherry-N1 (Clontech) as a template for mCherry (Supplementary Table 1).

To generate lentiviral expression vectors, the coding sequence of tagged or untagged β1 integrin, GFP or paxillin-mCherry was PCR amplified with primers designed according to the Gateway Cloning manufacturer’s instructions (ThermoFisher Scientific) to introduce flanking attB1 and attB2 recombination sites (Supplementary Table 1). pENTRY vectors were generated by Gateway BP recombination with pDONR221 and final lentivirus expression vectors were generated by Gateway LR recombination with pLENTI CMV Puro DEST (AddGene plamid #17452, gift from Eric Campeau). All constructs were verified by DNA sequencing.

### Cell culture and transfection

Immortalized β1 integrin floxed (β1 integrin fl/fl) and Cre-induced β1 integrin null (β1 integrin KO) fibroblasts^36^ were provided by Antony Koleske (Yale University) and shown to be mycoplasma free using MycoAlert (Lonza). β1 integrin KO cells were cloned by limited dilution and the absence of surface β1 integrin in the resulting clonal lines was verified by flow cytometry using a biotinylated anti-CD29 antibody (clone HMβ1-1 from Biolegend 112203). Fibroblasts, HEK 293T (a gift from Peter Tattersall, Yale University) and HeLa cells (a gift from Anton Bennett, Yale University) were cultured in Dulbecco modified Eagle medium (DMEM) high glucose containing glutamine supplemented with 9% bovine serum or fetal calf serum, non-essential amino acids and sodium pyruvate. Transfections were performed using PEI (Linear Polyethylenimine MW 25,000, Polysciences, Inc.).

### Lentivirus production and infection

Lentiviruses were produced by co-transfecting HEK293T cells with packaging vectors psPAX2 (viral proteins Gag and Rev under the SV40 promoter; Addgene plasmid #12260, a gift from D. Trono, École Polytechnique Fédérale de Lausanne, Lausanne, Switzerland) and pMD2.G (viral protein VSV-G expressed under the CMV promoter; Addgene plasmid #12259, a gift from D. Trono) together with the pLENTI CMV constructs. Viral supernatant was collected 48 and 72 h after transfection and filtered with a 0.45-µm filter. Cell lines were transduced by incubating with viral supernatant (diluted 1:2 to 1:16) and 8 µg ml^−1^ polybrene (Sigma) for 18 h, and selected with puromycin when necessary.

### Flow cytometry and analysis of integrin activation

Cell surface levels of human β1, mouse β1, and mouse α5 integrin were measured by flow cytometry on an LSRII instrument (BD Biosciences) using primary antibodies specific for human β1 integrin (P5D2 from Developmental Studies Hybridoma Bank, dilution 1:17; HUTS-4 from EMD Millipore MAB2079Z, dilution 1:1500), mouse β1 integrins (biotinylated anti-CD29 clone HMβ1-1 from Biolegend 112203, dilution 1:50), and mouse α5 integrin (clone MFR5 from EMD Millipore MABT18, dilution 1:100) followed by either APC-Steptavidin (ThermoFisher Scientific) or Alexa Fluor 647-conjugated anti-mouse or anti-rat secondary antibodies (Invitrogen, dilution 1:500). For control unstained cells, the incubation with primary antibody was omitted.

The activation state of endogenous α5β1 integrins was assessed using a modified version of a previously described flow cytometric assay^40^. Briefly, human FN repeats 9-10 fused to an N-terminal His-tagged Maltose-Binding Protein affinity tag, was purified from *E. Coli* and biotinylated with *N*-hydroxysuccinimidobiotin (ThermoFisher Scientific; FN9-10; manuscript in preparation). Mammalian cells were suspended with trypsin/EDTA, incubated with FN9-10 in the presence or absence of 10 mM EDTA or 0.5 mM MnCl_2_, fixed with 4% paraformaldehyde, incubated with APC-Strepatavidin and analyzed by flow cytometry on an LSRII instrument (BD Biosciences). The activation index of cells was calculated as AI = (*F*−*F*_o_)/*F*_integrin_, where *F* is the geometric mean fluorescence intensity (MFI) of FN9-10 binding, *F*_o_ is the MFI of FN9-10 binding in presence of inhibitor, and *F*_integrin_ is the normalized MFI of anti-β1 or anti-α5 integrin antibody binding to cells. Flow cytometry data were analyzed using FlowJo software. Data analysis and statistics were performed using GraphPad Prism.

### Adhesion assay

Cell adhesion assays in matrix-coated 96-well plates were based on published protocols^55^. Briefly, Costar flat bottom 96-well plates were coated with FN (0.1–10 μg ml^−1^, bovine plasma FN from Sigma) or type I collagen (1–10 μg ml^−1^, rat tail collagen from Gibco) and blocked with 10 mg ml^−1^ heat-denatured BSA (bovine serum albumin fraction V, American Bioanalytical). Cells were plated in replicate wells in serum-free DMEM and returned to 37 °C incubator for 30 min to 3 h. In these conditions, cell adhesion to control uncoated BSA-blocked wells was negligible. At the end of the assay, the wells were carefully washed with PBS, cells were fixed with 5% glutaraldehyde, stained with Crystal Violet, washed, and bound dye was resuspended in 10% acetic acid, as previously described^55^. Absorbance at 570 nm (A) was measured on a Tecan Infinite M200 microplate reader. Background signal (B) corresponded to absorbance in wells stained without cells. In a separate duplicate plate, cells were not washed prior to fixation/staining, and absorbance was used to determine “100% adhesion” (total A). Cell adhesion was calculated as (A–B)/(total A–B). Data analysis and statistics were performed using GraphPad Prism.

### Internalization assay

Assessment of integrin internalization was performed as previously described^56, 57^. Briefly, cell surface proteins were biotinylated on ice with 0.2 mg ml^−1^ NHS-SS-biotin (Pierce) on ice. After 5–30 min internalization at 37 °C, remaining surface biotin was removed with 50 mM Tris(2-Carboxyethyl) phosphine Hydrochloride (TCEP) and cells were lysed in PBS 0.5% Triton X-100. Lysates were clarified at 10,000 × *g* for 10 min and biotinylated proteins were pulled down with Streptavidin agarose beads (Sigma). Pulled-down β1 integrins were detected by immunoblotting with anti-CD29 antibody (clone 18 from BD Biosciences 610468, dilution 1:1000). A fraction of each bead supernatant was immunoblotted for β tubulin (clone E7 from Developmental Studies Hybridoma Bank, dilution 1:1000) as a loading control. The amount of β1 integrins internalized was calculated as a percentage of total surface signal.

### Immunoblotting

Pellets of fibroblasts were resuspended in Laemli Loading Buffer in reducing conditions, passed through a 23G needle 3 times and boiled for 5 min, fractionated by sodium dodecyl sulfate polyacrylamide gel electrophoresis and immunoblotted anti-β1 integrin (anti-CD29 clone 18 from BD Biosciences, dilution 1:1000), anti-GFP (Rockland 600-101-215, dilution 1:1000), or anti-β-tubulin (clone E7 from Developmental Studies Hybridoma Bank, dilution 1:1000) antibodies. After incubation with IRDye680 or IRDye800-conjugated secondary antibodies, the signal was scanned on an Odyssey CLx Imaging system, quantified and processed using Image Studio Lite (LI-COR Biosciences). Uncropped imaged are shown in Supplementary Figs. 10 and 11.

### Fluorescence imaging and immunofluorescence

Cells were plated on glass coverslips or glass-bottomed dishes (MatTek) coated with 10 μg ml^−1^ FN or 10 µg ml^−1^ type I collagen in phenol red-free DMEM culture medium. When specified, live cells were incubated with 250 nM HaloTag Alexa Fluor 488 (Promega) for 30 min at 37 °C. For live imaging, cells were incubated with Prolong Live anti-fade reagent (Invitrogen). Otherwise, for β1 integrin and vinculin immunofluorescence, cells were fixed for 30 min with 4% Paraformaldehyde in PBS 0.1% Triton X-100 or in cytoskeletal buffer (10 mM Mes,150 mM NaCl, 5 mM EGTA, 5 mM glucose, 5 mM MgCl_2_, and pH 6.1) and quenched/blocked with 50 mM NH_4_Cl, 0.2% BSA and 0.1% Triton X-100 in PBS. Cells were incubated with anti-β1 integrin antibodies (9EG7 from BD Biosciences 553715, dilution 1:300 or AIIB2 from Developmental Studies Hybridoma Bank, dilution 1:200) and an anti-vinculin antibody (hVin1 from Sigma V-9131, dilution 1:10,000), subsequently with Alexa Fluor 488, 568, or 647 secondary antibodies (Invitrogen, dilution 1:500). All fixed cells were mounted in ProLong Diamond anti-fade reagent (Invitrogen) visualized using a Nikon TI microscope under a ×100 immersion oil objective. For ER, endosome, or Golgi immunofluorescence, ecto-Halo β1 cells sequentially labeled with Alexa488 and SiR647 Halo ligands were incubated for 1 h at 37 °C after labeling, then washed with PBS, fixed for 20 s in 4% PFA, 0.2% Glutaraldehyde, and 0.1% Triton X-100 and for another 10 min in just 4% PFA and 0.1% Triton X-100. After blocking with 5% BSA PBS, 0.05% Tween 20, the cells were incubated with mouse monoclonal against SERCA2 (ER; Fisher Scientific MA3-919, dilution 1:300), GM130 (cis-Golgi; BD transduction 610823, dilution 1:300), or TfRc (endosomes; Life technologies 136800, dilution 1:300). The cells were then incubated with goat-anti mouse secondary antibody labeled with Alexa568 (dilution 1:1000). The samples were mounted as above and imaged on a Yokogawa-type spinning-disk confocal microscope (SDCM) as described below.

### Live-cell imaging

For total internal reflection fluorescence microscopy (TIRFM), ecto-pHluorin β1 integrin expressing cells were plated on glass-bottomed dishes (MatTek) coated with 10 μg ml^−1^ FN in phenol red-free DMEM supplemented with 9% bovine serum and 25 mM Hepes (imaging medium). Images were acquired on a microscope (IX-70; Olympus) equipped with 488 nm (150 mW) and 568 nm (100 mW) laser lines, temperature-controlled stage set (custom built), a TIRFM condenser (custom condenser), a 60 × 1.49 NA TIRF objective (Olympus), an EMCCD camera (iXion887; Andor Technology, pixel size = 189 nm), a pair of xy Galvo mirrors that are capable of switching from a TIRF light path to wide-field point-scanning bleaching illumination and is controlled with in-house C++ control software (developed by V. Polejaev, Yale University). Cells were imaged for 100 frames, photobleached for 2 s, and imaged by TIRFM again for 1500–2000 frames at a 6 frames per s frequency to detect fusion events for a total of 3 to 5 min. The 488 nm laser was adjusted to 50–60% of the power (~200 µW measured at the objective with a S170C microscope slide power sensor from Thorlabs). Detection and classification of fusion events was performed as described previously^43, 44^. Image analysis and enhancement were carried out with ImageJ.

To image endocytosis of ecto-Halo β1 in live cells, cells were labeled with 250 nM Alexa488 Halo ligand for 5 min, washed twice with medium at RT, and imaged live at 37 °C with a TIRFM microscope and HILO illumination. Images of 5 cells were acquired every 30 s for a total of 40 min. Surface fluorescence was then quenched with 5 µg ml^−1^ anti-Alexa488 antibody (Life technologies A11094) and cells were imaged for an additional 10 min. For co-localization with transferrin and the endosomal marker EEA1, Alexa488 Halo-labeled cells were incubated with 5 µg ml^−1^ Alexa568-transferrin (Tf568, Life technologies T23365) for 30 min in medium at 37 °C, fixed and stained for EEA1 (Cell signaling 3288T) by immunofluorescence as described above, and imaged by Wide-field illumination microscopy on an OMX microscope and deconvolution.

To label surface and internal pools of ecto-Halo β1 integrins, cells plated in FN-coated MatTek dishes were labeled sequentially with 250 nM HaloTag Alexa Fluor 488 (Promega) and 250 nM Halo-reactive SiR647-chloroalkane (SiR647-CA, gift from Promega) diluted in imaging medium for 15–30 min each. Cells were washed three times with imaging medium between and after labeling. The dynamics of sequentially labeled ecto-Halo β1 integrins at FA were monitored by TIRFM for 1 h in live cells at 37 °C or on cells fixed 0 or 1 h after labeling.

For 3D imaging, sequentially labeled ecto-Halo β1 integrin expressing cells plated on FN-coated MatTek dishes in imaging medium were imaged by confocal microscopy. We used a Yokogawa-type SDCM (Perkin-Elmer) mounted on an inverted microscope base (IX-71, Olympus) equipped with a 1 × 1 Kb electron-multiplying charge-coupled device camera (Hamamatsu Photonics), a temperature-controlled stage set (custom built), via a 60 × 1.4 NA oil objective lens with a pixel size of 143 nm using solid-state lasers: 488-, 640-nm (Melles Griot) with a 500 nm z-interval to image 8–10 µm of the cell volume. Acquisition and image analysis were performed using Volocity software (Perkin-Elmer).

### Spatial image analysis and modeling

To test whether fusion events occur at or near FAs, we computed the shortest distance from each fusion event to a FA, using a custom script written in MATLAB (Mathworks, Natick, MA, USA). Namely, this computation used the coordinates of each fusion event (561 fusion events from 5 cells; i.e., ~46–147 fusion events per cell) and corresponding binary masks identifying the position of FAs; the latter were generated using binary thresholding of the integrin channel prior to TIRFM photobleaching. For comparison, we simulated the same number of fusion events per cell (for each cell imaged) with random coordinates within the cell footprint, using Monte Carlo simulations (MATLAB plugin) for complete spatial randomness, and computed the shortest distance from these coordinates to FAs. This computation relied on binary masks identifying FAs (described above) while simulated coordinates of “fusion events” were randomly distributed within a manually defined binary mask identifying the cell. For each cell, this simulation was executed at least 100 times, yielding an average distance between randomly distributed coordinates and FAs. The real and synthetic data of each cell were plotted and compared by probability distribution functions.

### Data availability

All relevant data and MATLAB scripts are available from the authors upon request.

## Electronic supplementary material

Supplementary Information

Supplementary Movie 1

Supplementary Movie 2

Supplementary Movie 3

Supplementary Movie 4
