# Supplementary material for: Novel ecto-tagged integrins reveal their trafficking in live cells
Source: Nat Commun. 2017 Sep 18;8:570. doi: 10.1038/s41467-017-00646-w (PMC5603536; doi:10.1038/s41467-017-00646-w)
Supplement: Supplementary file 1 — Supplementary Information [file 41467_2017_646_MOESM1_ESM.pdf]

Description of Supplementary Files

File Name: Supplementary Information
Description: Supplementary Figures and Supplementary Table

File Name: Supplementary Movie 1
Description: A KO fibroblast reconstituted with ecto-pH4 β1 imaged for 3 min by TIRFM to illustrate integrin fusion events. Frames were taken every 100 ms, and are shown at 50 fps. Brightness, contrast and a Gaussian blur (0.75) on ImageJ were applied to improve the raw images appearance.

File Name: Supplementary Movie 2
Description: A HeLa cell expressing ecto-pH4 β1 imaged for 6 min by TIRFM to illustrate integrin fusion events. Frames were taken every 100 ms, and are shown at 50 fps. Brightness, contrast and a Gaussian blur (0.75) on ImageJ were applied to improve the raw images appearance.

File Name: Supplementary Movie 3
Description: A KO fibroblast reconstituted with ecto-Halo β1 integrin was labeled with Alexa488 Halo ligand (green), imaged for 40 min by HILO TIRFM. After 40 min, surface fluorescence was quenched with Anti-Alexa488 antibody to visualize integrins internalized in live cells. Frames were taken every 30 s, and are shown at 15 fps. Brightness, contrast and a Gaussian blur (0.75) on ImageJ were applied to improve the raw images appearance.

File Name: Supplementary Movie 4
Description: A KO fibroblast reconstituted with ecto-Halo β1 integrin was sequentially labeled with Alexa488 (green) and SiR647 (red) Halo ligands and imaged for 30 min by TIRFM to illustrate integrin dynamics at FAs. Frames were taken every 30 s, and are shown at 15 fps. Brightness, contrast and a Gaussian blur (0.75) on ImageJ were applied to improve the raw images appearance.

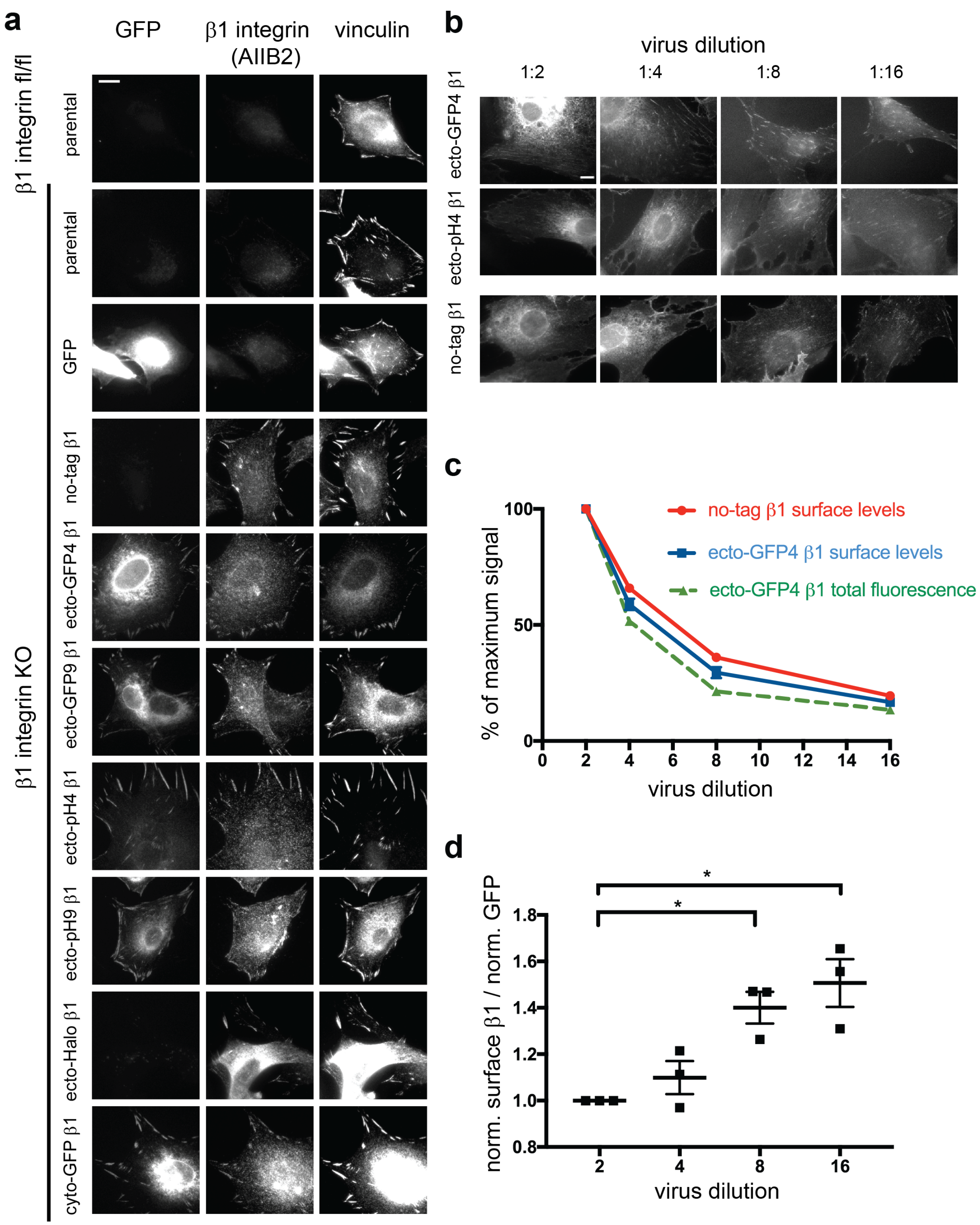

Supplementary Figure 1: No-tag and ecto-tagged β1 integrins accumulate inside the cells as a result of overexpression. (a) Microscopy images of parental and reconstituted fl/fl and KO fibroblasts showing GFP epifluorescence (left column), total β1 integrin immunofluorescence using AIIB2 antibody (center column), and vinculin immunofluorescence (right column). (b) Microscopy

images of a subset of reconstituted KO fibroblasts infected with 4 different dilutions of lentivirus showing GFP epifluorescence (ecto-GFP4 β1 and ecto-pH4 β1 cells, top panel) or total β1 integrin immunofluorescence using AIIB2 antibody (no-tag β1 cells, bottom panel). Scale bar, 10 μm. (c) Quantification of surface levels of human β1 integrin and total GFP fluorescence by flow cytometry on a subset of reconstituted β1 integrin KO fibroblasts infected with 4 different dilutions of lentivirus. Data is shown as Mean +/- SEM from 3 independent experiments. (d) Ratio between normalized surface β1 integrin levels and normalized GFP fluorescence quantified by flow cytometry on KO cells infected with 4 different dilutions of ecto-GFP4 β1 expressing-lentivirus. Data is shown as Mean +/- SEM from 3 independent experiments. Paired comparisons were performed with two-tailed paired Student *t*-test and * indicates $p<0.05$.

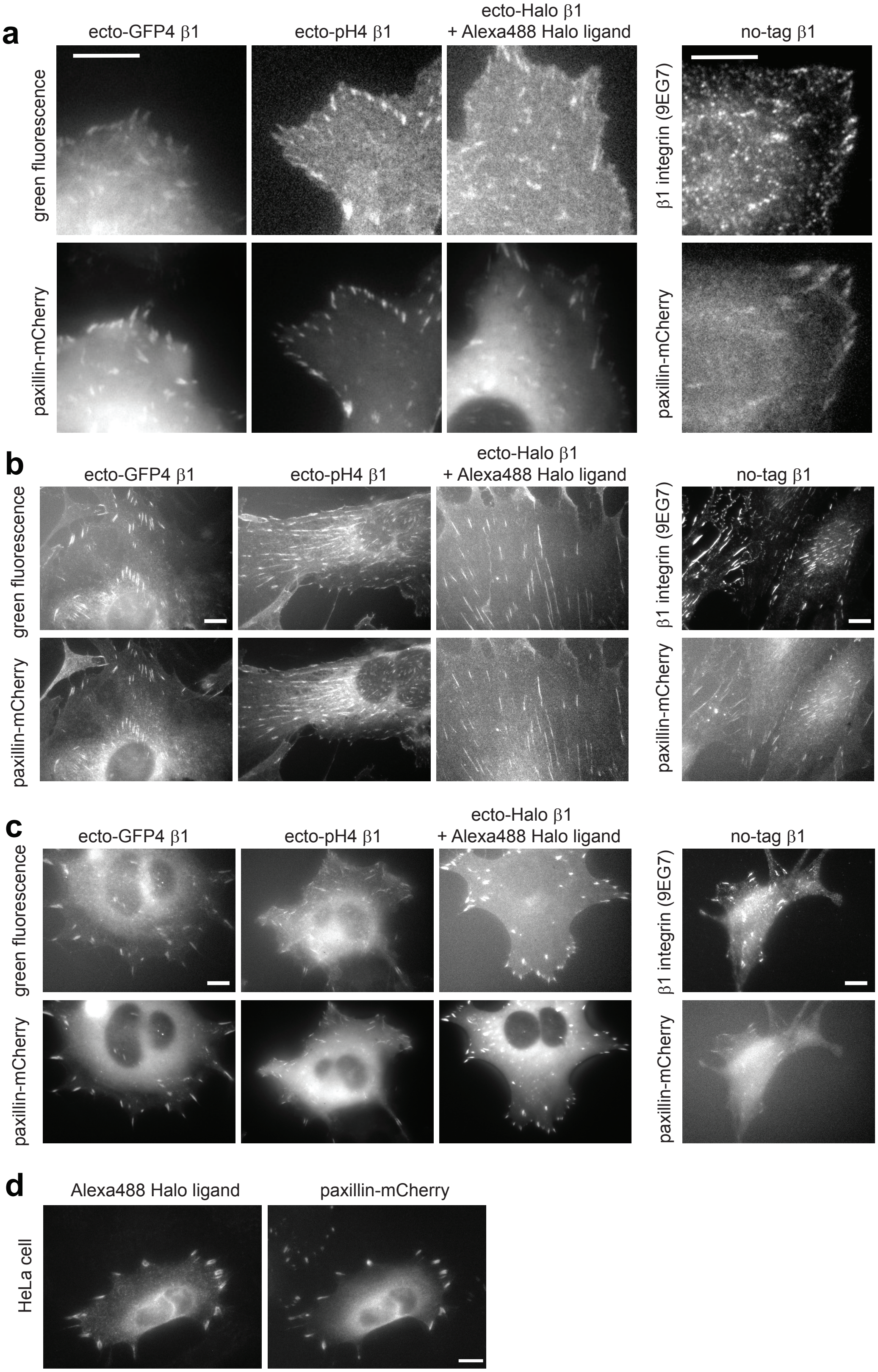

Supplementary Figure 2: Ecto-tagged β1 integrins target to various kinds of adhesions. (a-d) Microscopy images showing co-localization of paxillin-mCherry with ecto-tagged and no-tag β1

integrins in reconstituted KO fibroblasts (a-c) or HeLa cells (d), Nascent adhesions formed after 1 h on FN (a), mature and fibrillar adhesions formed after 24 h on FN (b), and FAs formed after 3 h on type I collagen in DMEM 0.1% FBS (c) or 20 h on FN (d). Ecto-GFP4 β1, ecto-pH4 β1 integrins and paxillin-nCherry were visualized by their green epifluorescence, ecto-Halo β1 integrins were labeled with the cell-impermeant HaloTag® Alexa Fluor® 488 ligand, and no-tag β1 integrins were visualized by immunofluorescence using 9EG7 antibody. Imaging was performed on fixed cells except for a, left panel in which imaging was done on live cells. Scale bar, 10 μm.

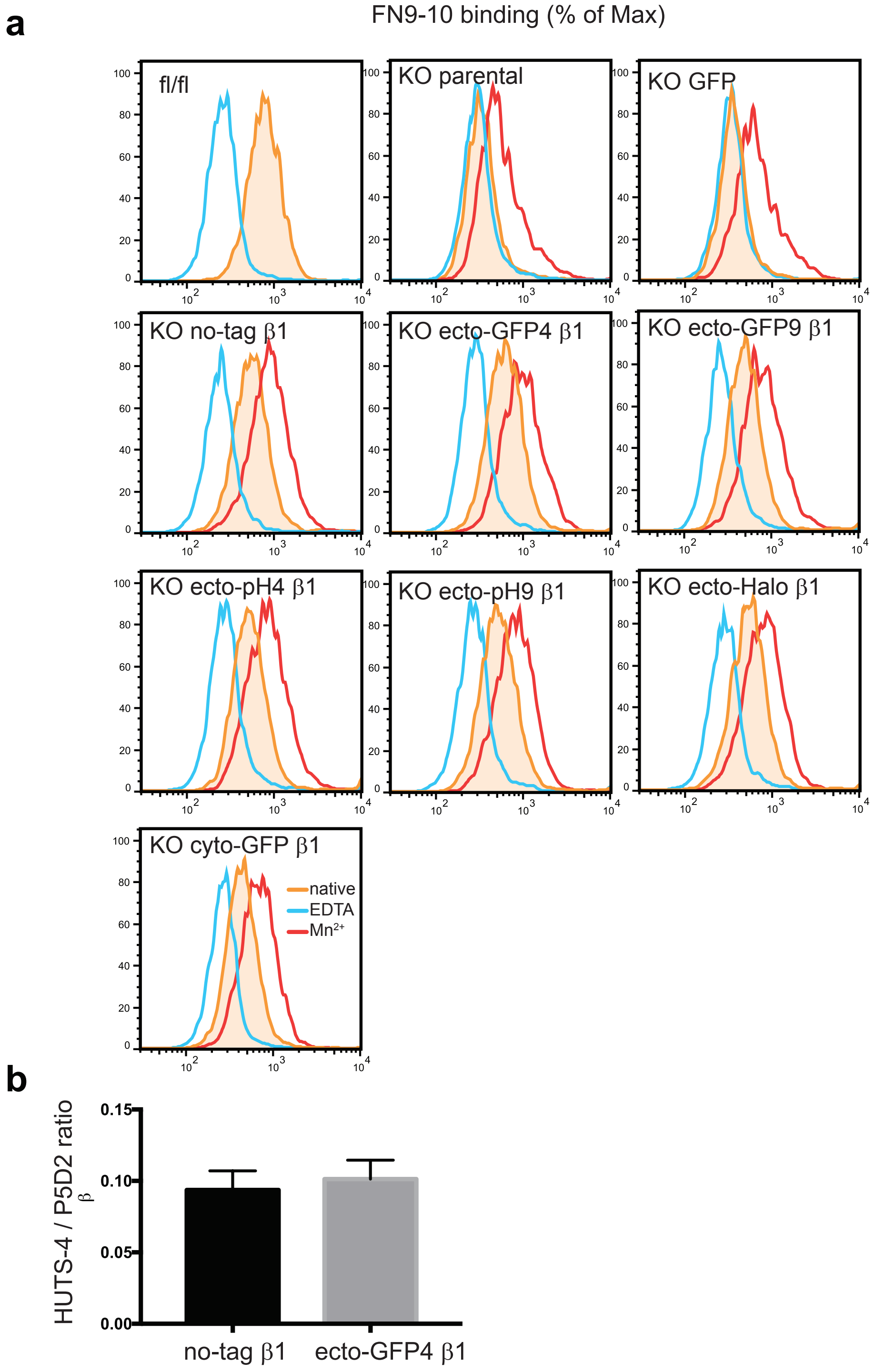

Supplementary Figure 3: Additional flow cytometry data. (a) Histograms showing binding of soluble FN9-10 to parental and all reconstituted β1 integrin fl/fl and KO fibroblasts, in native conditions (filled orange peak), EDTA-inhibited conditions (blue peak), or Mn^{2+}-treated conditions (red peak). (b) Ratio of HUTS-4 binding and P5D2 binding to KO fibroblasts reconstituted with either no-tag β1 or ecto-GFP4 β1 integrin. Data is shown as Mean +/- SEM from 3 independent experiments.

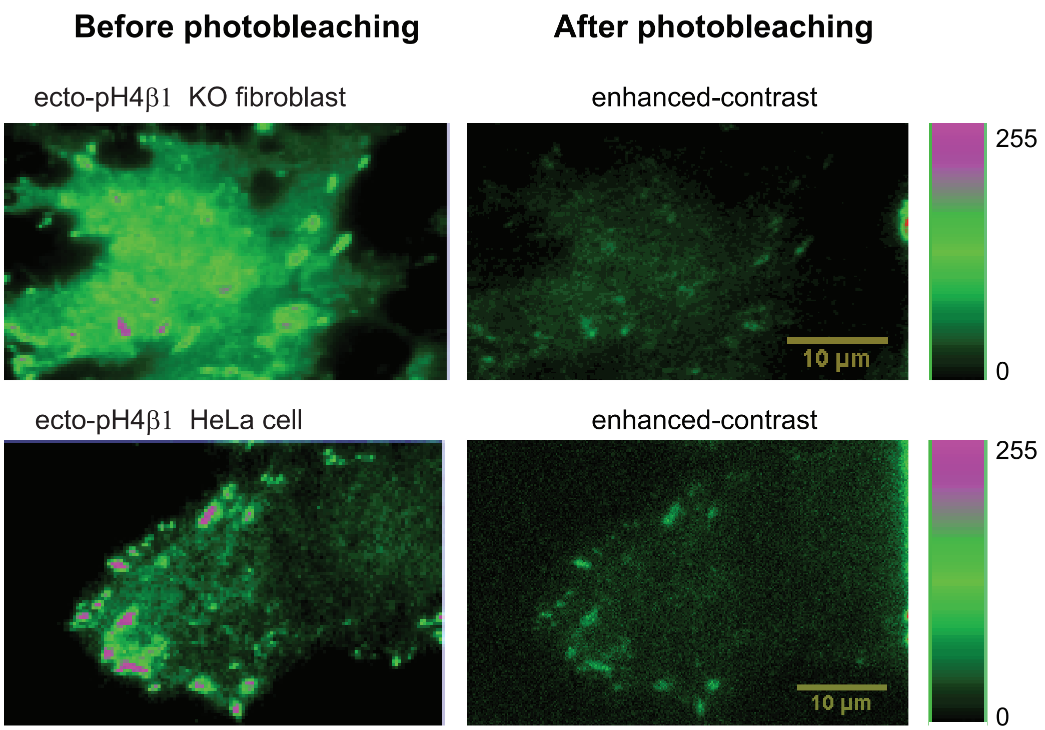

Supplementary Figure 4: Surface ecto-pH4 β1 integrin signal in a KO fibroblast and a HeLa cell before and after photobleaching during TIRFM imaging facilitated detection of integrin fusion events.

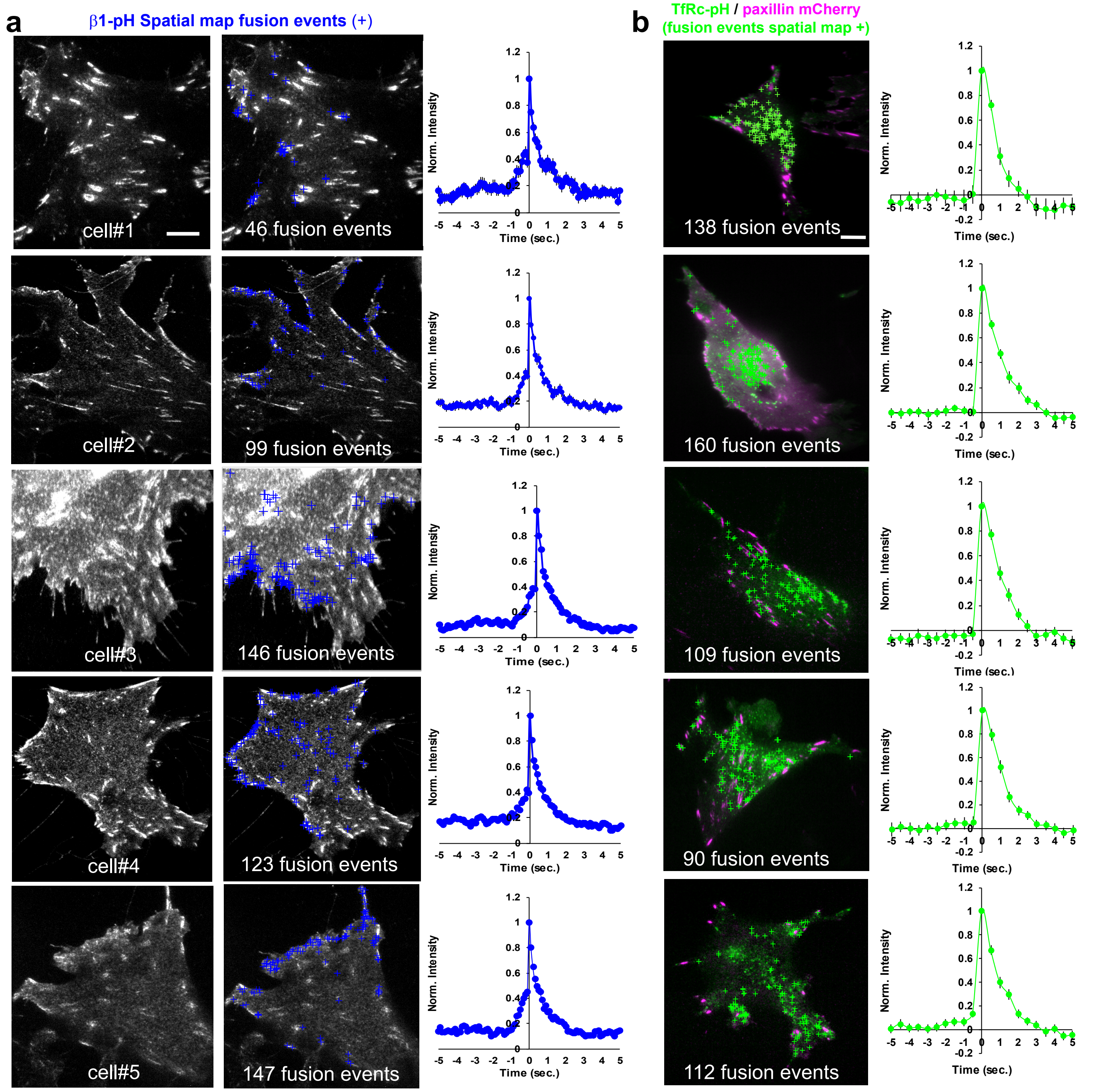

Supplementary Figure 5: (a) Spatial map (blue crosses) and the temporal alignment (line graph; Mean +/- SEM) of fusion events detected on 5 KO fibroblasts reconstituted with ecto-pH4 β1 integrin that were used to quantify the distance of fusion events to the nearest FA. Scale bar, 10 μm. (b) Spatial map (green crosses) and the temporal alignment (line graph; Mean +/- SEM) of fusion events detected on 5 KO fibroblasts expressing Transferrin Receptor-pHluorin (TfRc-pH, green) and paxillin-mCherry (magenta) that were used to quantify the distance of TfRc-pH fusion events to the nearest FA. Scale bar, 10 μm.

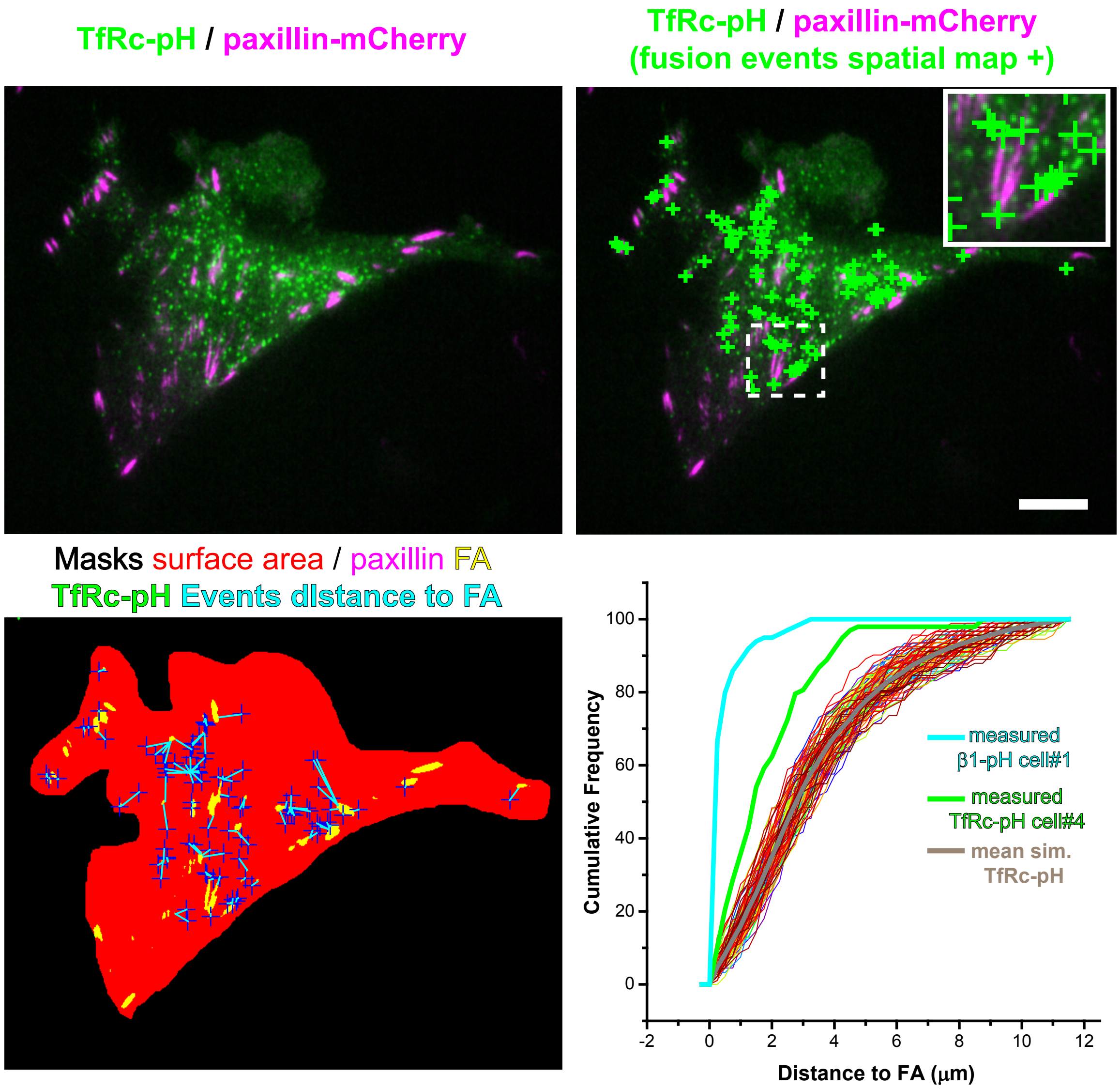

Supplementary Figure 6: Spatial distribution analysis of TfRc-pH fusion events relative to paxillin m-Cherry labeled FAs. Top left panel, merged image of a fibroblast expressing TfRc-pH (green) and paxillin-mCherry (magenta). Top right panel, distribution of fusion events (green crosses) along the cell and FAs (dash square, inset). Scale bar, 10 µm. Bottom left panel, the distance of TfRc-pH fusion events to the nearest paxillin-mCherry labeled FA (yellow mask) was measured (cyan lines). Right bottom graph, Cumulative frequency chart demonstrating the difference in distances to FA between the measured β1-pH data (cyan line), the measured TfRc-pH data (green line), and 100 simulations for the TfRc-pH events (individual simulation, color lines; mean, gray line) that were used to calculate the median distances used on Fig. 6c.

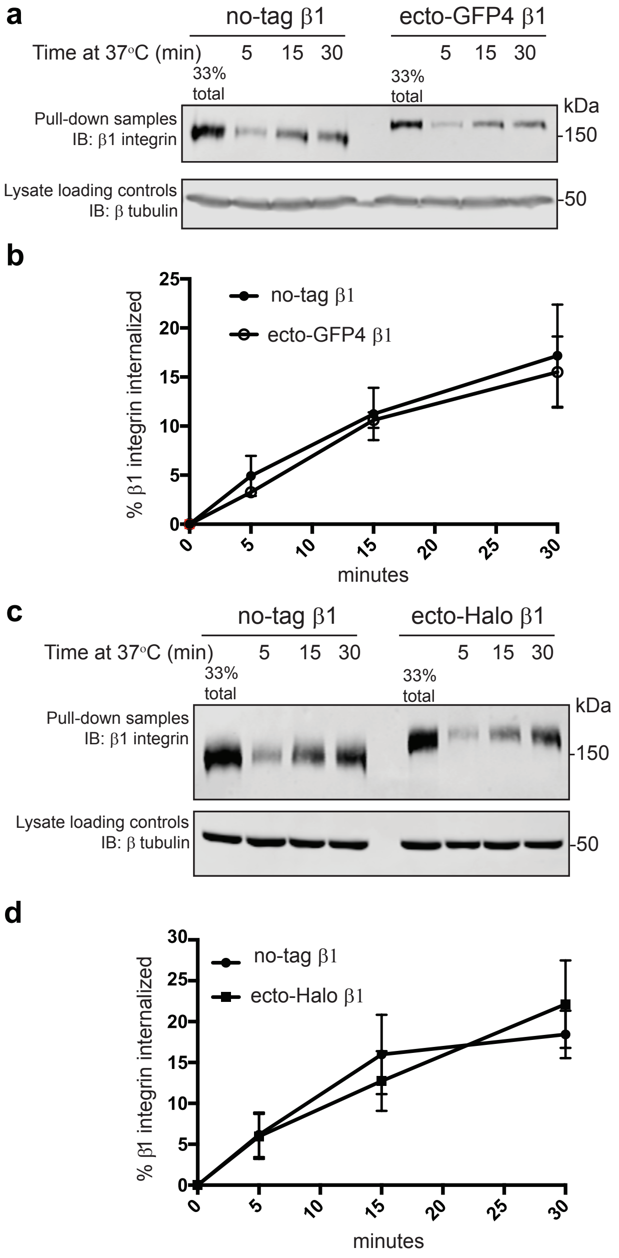

Supplementary Figure 7: Untagged and ecto-tagged β1 integrins internalize at the same rate. (a) and (c), Reversible surface biotinylation assay showing β1 integrins (a, no-tag and ecto-GFP4; c, no-tag and ecto-Halo) internalized after 5, 15 or 30 min at 37°C, and total surface β1 integrins (33% loaded), pulled down by Streptavidin-agarose and detected by immunoblotting. Lysate loading controls (2.5%) were blotted for β tubulin to demonstrate equal loading. (b) and (d), Quantification of the signal corresponding to internalized β1 integrins after 5, 15 or 30 min at 37°C. Data correspond to Mean +/- SEM from 5 independent experiments. Uncropped blots are shown in Supplementary Fig. 11.

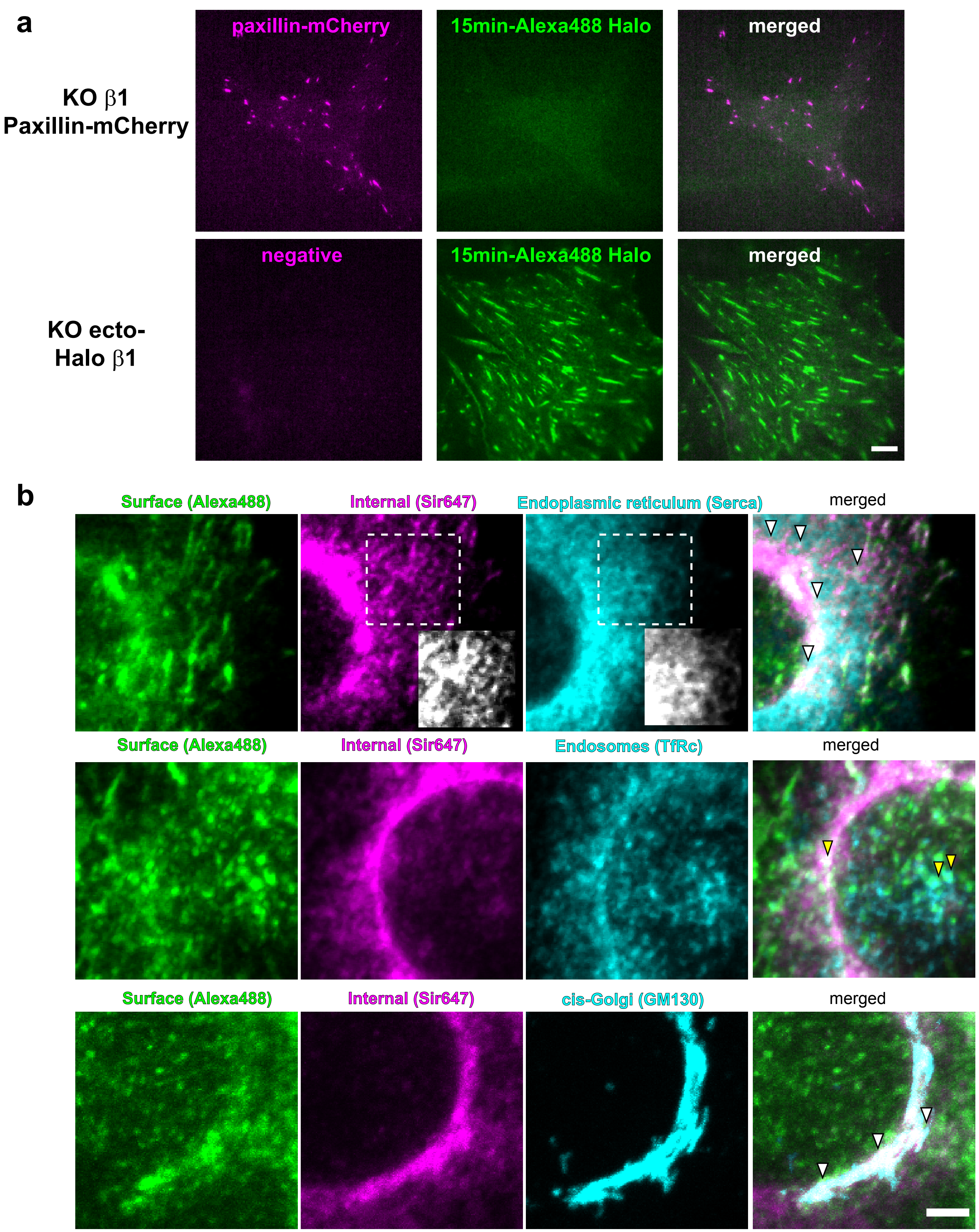

Supplementary Figure 8: Specificity and co-localization analysis of ecto-Halo β1 integrin labeling. (a) Live cells HILO TIRFM imaging of KO fibroblasts reconstituted with untagged β1 integrin

and paxillin-mCherry (top row, magenta) or KO fibroblasts reconstituted with ecto-Halo β1 integrin (bottom row, green) demonstrating that, after a 15 min incubation with Alexa488 Halo ligand, only the ecto-Halo β1 fibroblasts accumulate Alexa488 at their FAs. (b) Immunofluorescence analysis by confocal microscopy of markers of ER (Serca), endosome (TfRc) and cis-Golgi (GM130) in KO fibroblasts expressing ecto-Halo β1 integrins, subjected to sequential labeling of surface integrins with Alexa488 Halo ligand and internal integrins with Sir647 Halo ligand and incubated for 1 h at 37°C after labeling. Internally labeled SiR647 ecto-Halo β1 integrins (magenta) preferentially co-localized with ER (white arrowheads top panel) on ER tubules (gray insets from dashed line square region) and with the cis-Golgi (white arrowheads bottom panel) demonstrating trafficking of ecto-Halo β1 integrins along the secretory pathway. Surface labeled Alexa488 ecto-Halo β1 integrins (green) showed partial co-localization with the endosomes (yellow arrowheads middle panel) demonstrating endocytosis of surface ecto-Halo β1 integrins.

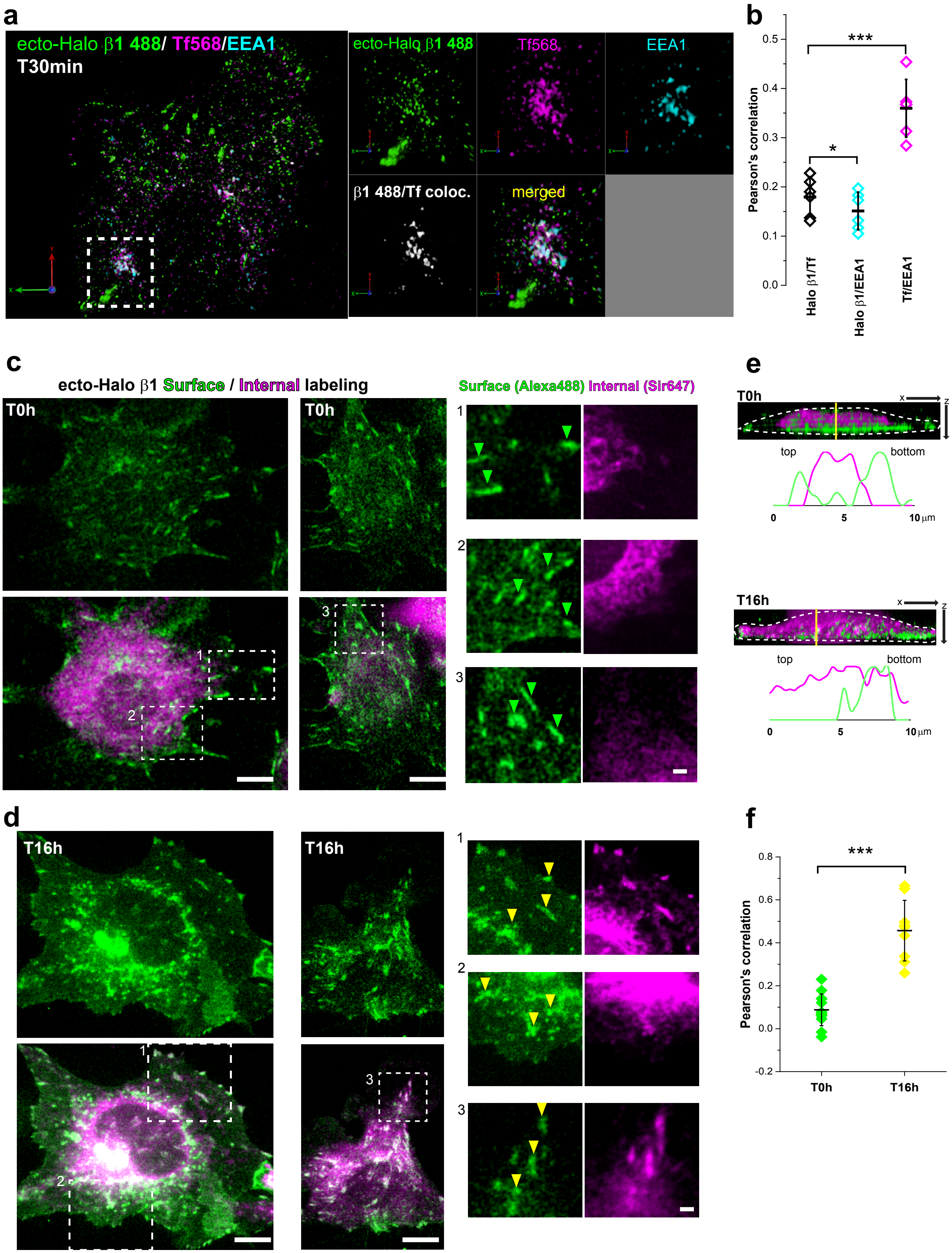

Supplementary Figure 9: Selective surface and internal labeling of ecto-Halo β1 integrins demonstrates integrin endocytosis in live cells. (a) Overlay images of KO fibroblasts reconstituted

with ecto-Halo β1 integrins, labeled for 5 min with Alexa488 Halo ligand (ecto-Halo β1 488, green), then for 30 min with Transferrin-Alexa568 (Tf568, magenta), fixed, stained for EEA1 by immunofluorescence (EEA1, cyan) and imaged by OMX wide-field illumination microscopy and deconvolution. Images on the right show a detailed region of the cell (dashed line) for each labeling, an overlay of the ecto-Halo β1 488 and the Tf568 signals (β1 488/Tf568 co-localization, white image) and a merged image of all 4 channels. (b) Pearson's correlation analysis of quantified signals demonstrating a significantly higher co-localization between internalized ecto-Halo β1 integrins and internalized transferrin (Halo β1/Tf) than between internalized β1 and EEA1 (Halo β1/EEA1). The scatter-box graph showed the Mean +/- SD for 5 cells for each analysis. Statistical analysis was performed using a two samples Student *t*-test and * indicates $p<0.05$, or *** indicates $p<0.001$. (c-d) Overlay images of KO fibroblasts reconstituted with ecto-Halo β1 integrins, immediately after (c, left panel) and 16 h after (d, left panel) sequential labeling with cell-impermeant Alexa488 Halo Ligand (Surface, green label) and cell-permeant SiR647 Halo Ligand (Internal, magenta label), imaged by Spinning-Disk Confocal microscopy on live cells. Scale bar, 15 µm. Images on the right show details of the labeling with each dye in three regions of the cell (dashed line square). Green arrowheads indicate green-only FAs at T0 and yellow arrowheads indicate co-localization of the dyes in FAs and internal compartments 16h after labeling. Scale bar, 5 µm. (e) *x-z* volume profiles of the labeled cells (cell outline, dashed lines) illustrating the re-distribution of the external (green label) and internal (magenta label) integrins before and after the 16 h incubation. Line graphs represent the intensity line profiles analysis along the *z*-axis (yellow line) showing the increased co-localization after incubation. (f) Pearson's correlation analysis between Alexa488 and SiR647 signals demonstrating the significant increase in co-localization after 16 h incubation. The scatter-box graph showed the Mean +/- SD for 13 and 9 cells respectively. Statistical analysis was performed using a two samples Student *t*-test and *** indicates $p<0.001$.

b

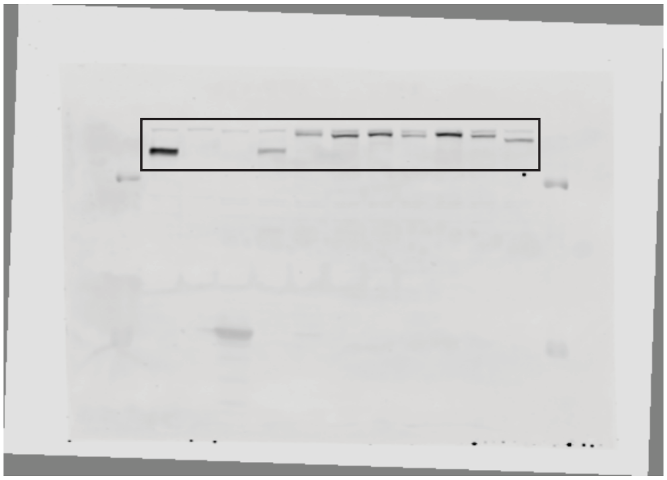

IB: β1 integrin

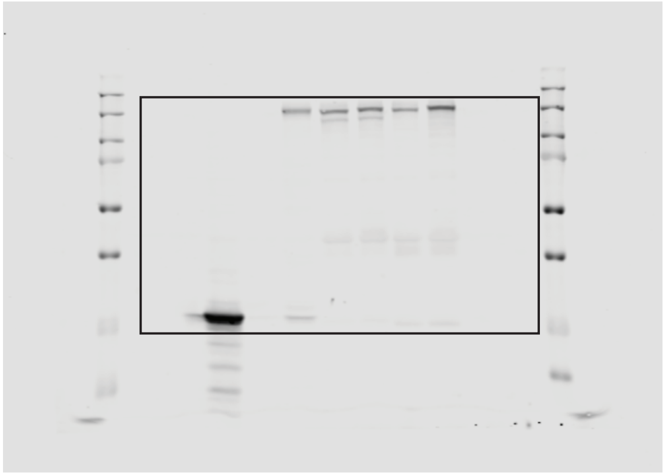

IB: GFP

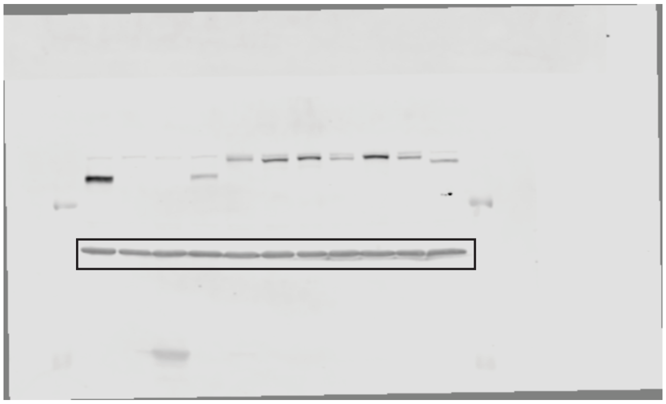

IB: β-tubulin

Supplementary Fig. 10: uncropped blots from Fig. 2

a

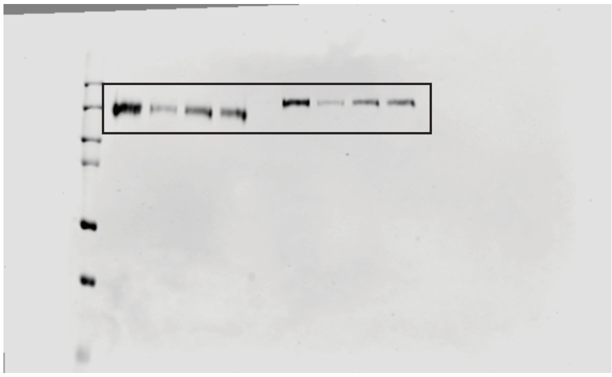

IB: β1 integrin

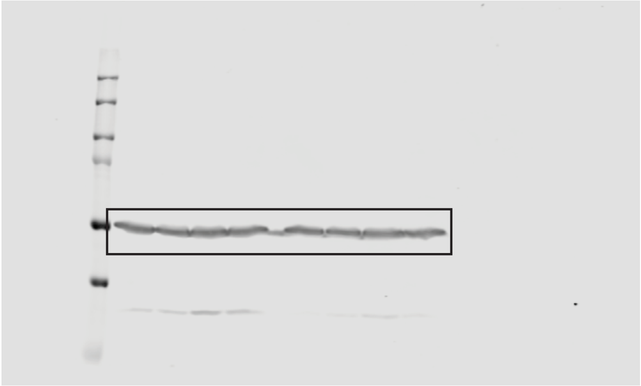

IB: β-tubulin

c

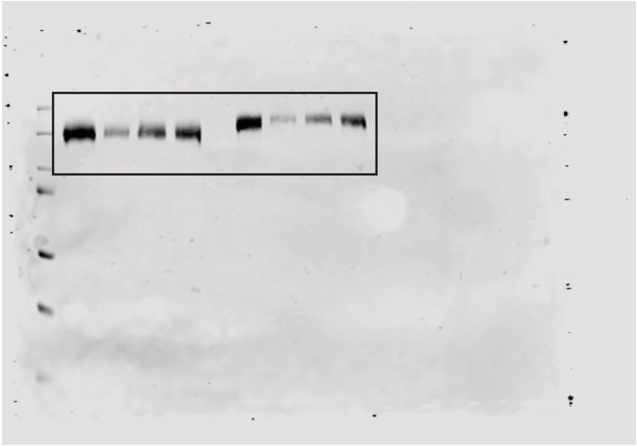

IB: β1 integrin

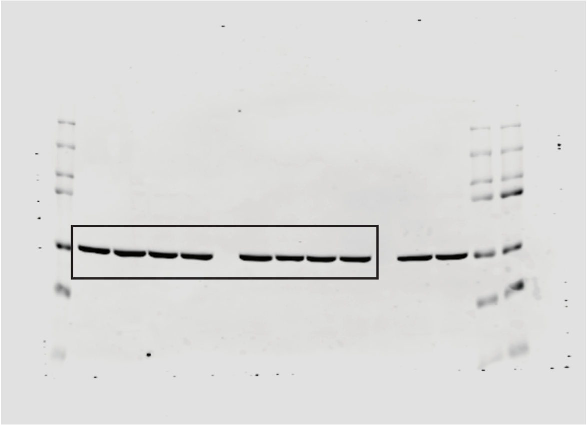

IB: β-tubulin

Supplementary Fig. 11: uncropped blots from Supplementary Fig. 7

**Primer**	**Primer sequence (5’-3’)**
β1 integrin EcoRI-XhoI For	GTAACCAACCGTAGCAAAGGAGAATTCCTCGAGACAGCAGAGAAGCTCAAGCCAGAG
β 1 integrin EcoRI-XhoI Rev	CTCTGGCTTGAGCTTCTCTGCTGTCTCGAGGAATTCTCCTTTGCTACGGTTGGTTAC
4AA GFP EcoRI For	GAATTCGGAGGTATGGTGAGCAAGGGC
4AA GFP XhoI Rev	CTCGAGACCTCCCTTGTACAGCTCGTC
9AA GFP EcoRI For	GAATTCGGAGGTTCTGGAGGTTCTGGTATGGTGAGCAAGGGC AG
9AA GFP XhoI Rev	CTCGAGACCAGAACCTCCAGAACCTCCCTTGTACAGCTCGTC
4AA pHluorin EcoRI For	GAATTCGGAGGTATGAGTAAAGGAGAAG
4AA pHluorin XhoI Rev	CTCGAGACCTCCACTAGTTTTGTATAGTTCATCC
9AA pHluorin EcoRI For	GAATTCGGAGGTTCTGGAGGTTCTGGTATGAGTAAAGGAGAAGAAC
9AA pHluorin XhoI Rev	CTCGAGACCAGAACCTCCAGAACCTCCACTAGTTTTGTATAGTTCATCC
9AA Halo EcoRI For	GGGGAATTCGGAGGTTCTGGAGGTTCTGGTGAAATCGGTACTGGCTTTCCATTC
9AA Halo XhoI Rev	GGGCTCGAGACCAGAACCTCCAGAACCTCCACCGGAAATCTCCAGAGTAGACAG
9AA SNAP EcoRI For	GGGGAATTCGGAGGTTCTGGAGGTTCTGGTATGGACAAAGACTGCGAAATGAAG
9AA SNAP XhoI Rev	GGGCTCGAGACCAGAACCTCCAGAACCTCCCACCACCCGGTGGCAGGGGATCAG
attB1 β1 integrin For	GGGGACAAGTTTGTACAAAAAAGCAGGCTTCGGTACCATGAATTTACAACCAATTTTCTGG
attB2 β1 integrin Rev	GGGGACCACTTTGTACAAGAAAGCTGGGTCTAACTATTTTCCCTCATACTTCGGATT
attB1 paxillin-mCherry For	GGGGACAAGTTTGTACAAAAAAGCAGGCTTCGGTACCATGGACGACCTCGATGCCTTACTG
attB2 GFP/mCherry Rev	GGGGACCACTTTGTACAAGAAAGCTGGGTCCTATTACTTGTACAGCTCGTCCATGCC

Supplementary Table 1: Primers used to generate constructs.
